# *Plasmodium vivax* serological exposure markers: PvMSP1-42-induced humoral and memory B-cell response generates long-lived antibodies

**DOI:** 10.1371/journal.ppat.1012334

**Published:** 2024-06-28

**Authors:** Feng Lu, Jiahui Xu, Yaobao Liu, Zhenyu Ren, Junhu Chen, Weijuan Gong, Yi Yin, Yinyue Li, Li Qian, Xinlong He, Xiu Han, Zhijie Lin, Jingyuan Lu, Wenwen Zhang, Jiali Liu, Didier Menard, Eun-Taek Han, Jun Cao

**Affiliations:** 1 Department of Pathogenic Biology and Immunology, School of Medicine, Key laboratory of Jiangsu province university for Nucleic Acid & Cell Fate Manipulation, Affiliated Hospital of Yangzhou University, Yangzhou University, Yangzhou, China; 2 National Health Commission Key Laboratory of Parasitic Disease Control and Prevention, Jiangsu Provincial Key Laboratory on Parasite and Vector Control Technology, Jiangsu Provincial Medical Key Laboratory, Jiangsu Institute of Parasitic Diseases, Wuxi, China; 3 National Institute of Parasitic Diseases, Chinese Center for Disease Control and Prevention, Shanghai, China; 4 Institut Pasteur, Université Paris Cité, Malaria Parasite Biology and Vaccines Unit, Paris, France; 5 Université de Strasbourg, UR 3073—Pathogens Host Arthropods Vectors Interactions Unit, Malaria Genetics and Resistance Team (MEGATEAM), Strasbourg, France; 6 CHU Strasbourg, Laboratory of Parasitology and Medical Mycology, Strasbourg, France; 7 Department of Medical Environmental Biology and Tropical Medicine, School of Medicine, Kangwon National University, Chuncheon, Republic of Korea; University of Sao Paulo, BRAZIL

## Abstract

*Plasmodium vivax* serological exposure markers (SEMs) have emerged as promising tools for the actionable surveillance and implementation of targeted interventions to accelerate malaria elimination. To determine the dynamic profiles of SEMs in current and past *P*. *vivax* infections, we screened and selected 11 *P*. *vivax* proteins from 210 putative proteins using protein arrays, with a set of serum samples obtained from patients with acute *P*. *vivax* and documented past *P*. *vivax* infections. Then we used a murine protein immune model to initially investigate the humoral and memory B cell response involved in the generation of long-lived antibodies. We show that of the 11 proteins, especially C-terminal 42-kDa region of *P*. *vivax* merozoite surface protein 1 (PvMSP1-42) induced longer-lasting long-lived antibodies, as these antibodies were detected in individuals infected with *P*. *vivax* in the 1960-1970s who were not re-infected until 2012. In addition, we provide a potential mechanism for the maintenance of long-lived antibodies after the induction of PvMSP1-42. The results indicate that PvMSP1-42 induces more CD73^+^CD80^+^ memory B cells (MBCs) compared to *P*. *vivax* GPI-anchored micronemal antigen (PvGAMA), allowing IgG anti-PvMSP1-42 antibodies to be maintained for a long time.

## Author summary

**Fig 1:**
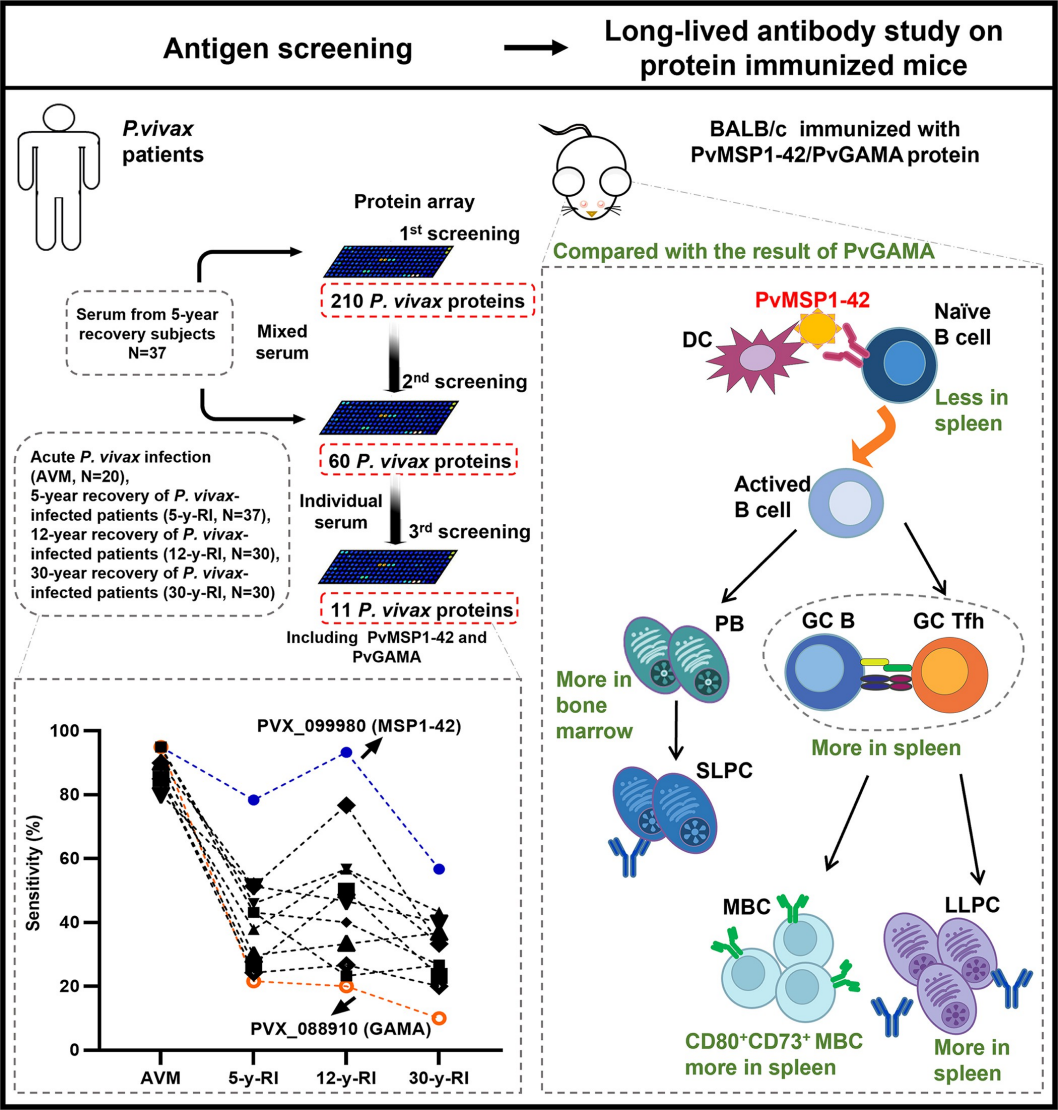
Summary diagram of experimental results.

*P*. *vivax* serological exposure markers (SEMs) that reflect exposure to blood-stage *P*. *vivax* parasites, could play an important role in assessing progress towards malaria elimination. Serological surveillance can be a useful tool for identifying areas of high transmission intensity or hidden asymptomatic reservoirs, especially as malaria transmission declines. These immune responses can persist long after the clearance of blood-stage infection, making antibodies valuable markers for both past exposure and current infection status. Furthermore, gaining a better understanding of the host immune response to malaria, particularly regarding the induction and maintenance of high levels of circulating antibodies, is crucial for effective serological surveillance efforts. Using serum obtained in 2007 or 2012 from patients with acute *P*. *vivax* infection, 5-year, 12-year and 30-year recovery of *P*. *vivax*-infected patients we provide evidence that PvMSP1-42 induces long-lived anti-PvMSP1-42 antibodies. Then we used a murine protein immune model, show that more CD73^+^CD80^+^ memory B cells, allowing IgG anti-PvMSP1-42 antibodies to be maintained for a long time (Fig 1). The results of this study represent an important step forward in our understanding of the host immune response to *P*. *vivax* blood stages, including the induction and maintenance of high levels of circulating antibodies. These findings may have important implications for the development of effective serological surveillance tools.

## Introduction

With an estimated of 249 million malaria cases in 2022, *Plasmodium vivax* malaria accounts for 2.8% of these, and remains a major threat to public health [1]. *P*. *vivax*, which is the most geographically widespread human malaria parasite [2], exhibits specific biological features that render its control more challenging than *falciparum* malaria [3]. These include the production of blood-stage gametocytes prior the onset of the malaria clinical symptoms and dormant liver-stage hypnozoites that can reactivate weeks to months after initial infection, leading to relapses that contribute to morbidity and perpetuate transmission [4]. As a result of the decline in malaria transmission over the past decade, it has become increasingly difficult to estimate the incidence of clinical *P*. *vivax*, mainly due to the poor performance of routine passive case finding in detecting asymptomatic carriers and submicroscopic infections [5]. To address the unique challenge posed by *P*. *viva*x to malaria elimination, we therefore need to develop reliable surveillance tools, such as *P*. *vivax* serological exposure markers (SEMs) which can be used as a sensitive measure of transmission [6]. Blood-stage *P*. *vivax* infection typically elicits a strong IgG antibody response to multiple proteins, even in low-density asymptomatic infections [7]. These antibody responses used as SEMs can persist for a long time, after *P*. *vivax* blood-stages have been cleared [8,9]. SEMs are therefore both a marker of current and past infection [8]. Knowledge of malaria transmission intensity is key information necessary to guide deployment of interventions and to monitor their effectiveness [10–12]. This is particularly important when trying to assess exposure to malaria in the context of elimination strategies. Previous work has indicated that serological assays can be efficiently used for this purpose as anti-malarial antibodies reflect exposure in the human host [13]. Due to the limited availability of *P*. *vivax* serum samples, studying the long-lasting humoral immunity of malaria presents a significant challenge. Consequently, few studies have focused on investigating the persistence of these SEMs.

Maintenance of antibody levels is known to be associated with the development of memory B cells (MBCs) and long-lived plasma cells (LLPCs) [14,15]. After that naïve B cells in secondary lymphoid organs bind and internalize antigen, the initial B cells are activated, and the humoral immune response begins [16]. Recognition of the antigen by the B cell receptor (BCR) initiates a series of signal transduction and gene expression changes that promote B cell trafficking, antigen presentation, and interaction with CD4^+^ helper T cells, known as T follicular helpers (Tfh) [17]. Following interaction with Tfh cells and subsequent stimulation by co-stimulatory molecules and cytokines, activated B cells can differentiate into at least two cell populations with distinct fates and functions: the plasmablasts (PBs), known as short-lived plasma cells (SLPCs), and the germinal center B cells (GC B), which further differentiation into MBCs or LLPCs [18,19]. LLPCs maintain long-term production of antibodies, that migrate to the microenvironment within the bone marrow [9,20] and spleen [21,22] and can persist in mice or humans [23–25]. Therefore, a better understanding of the host immune response to *P*. *vivax* blood stages, particularly how high levels of circulating antibodies are induced and maintained, is essential for the development of effective serological surveillance.

In this study, we used clinical serum samples from patients with current and past infections and screened 210 blood-stage proteins to identify *P*. *vivax* SEMs that may reflect past and current infections. To better understand the host immune response to *P*. *vivax* blood stages, such as how high levels of circulating antibodies are induced and maintained, we also investigated the potential factors involved in the antigen-induced production of long-lived antibodies.

## Results

### Patient clinical serum samples and production of recombinant blood-stage *P*. *viva*x proteins

**Fig 2:**
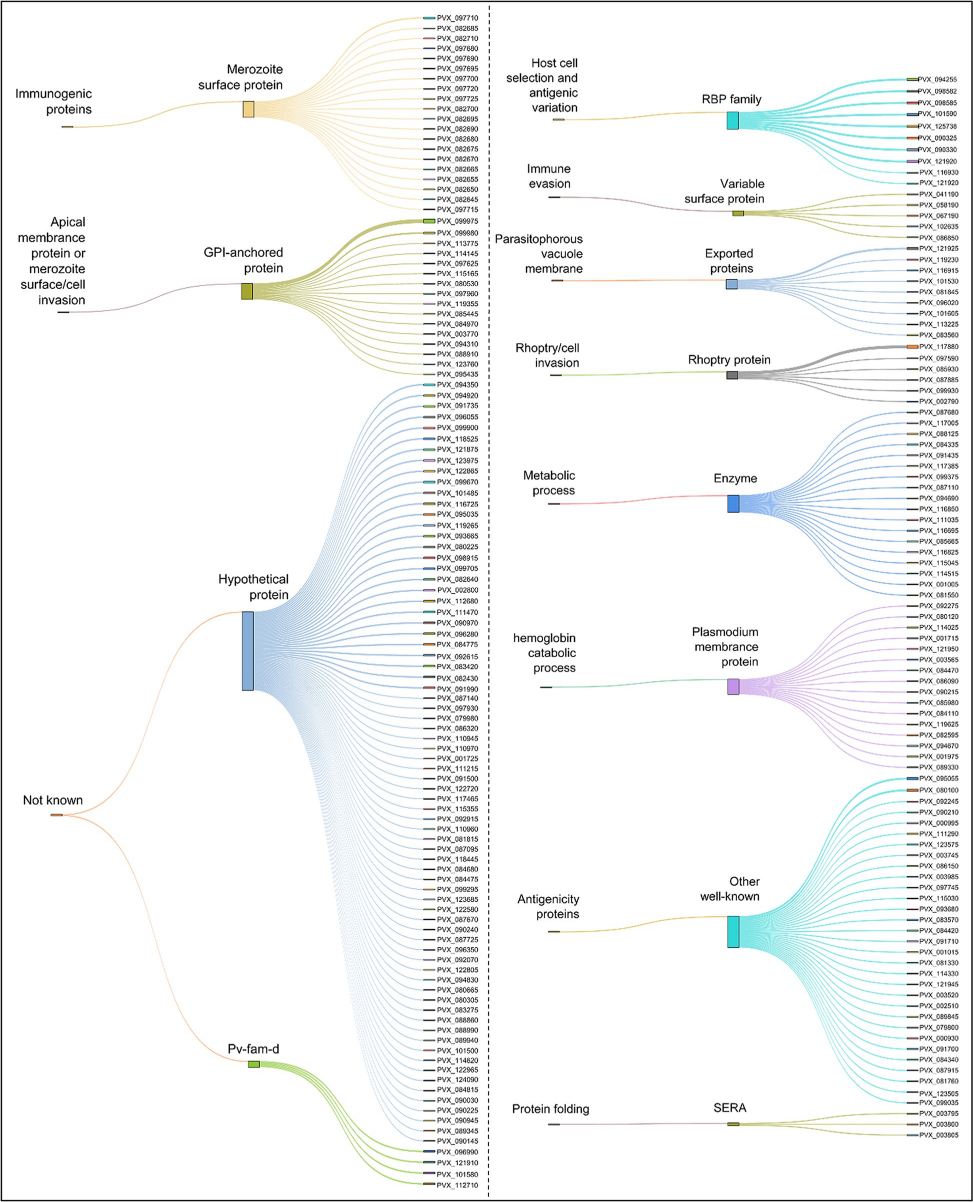
Classification of the two hundred and ten *P*. *vivax* blood-stage proteins expressed using the wheat germ cell-free (WGCF) method based on predicted subcellular functions or locations.

One hundred and thirty-seven serum samples were obtained in 2007 or 2012 from patients with acute *P*. *vivax* infection (AVM, N = 20), 5-year recovery of *P*. *vivax*-infected patients (5-y-RI, N = 37), 12-year recovery of *P*. *vivax*-infected patients (12-y-RI, N = 30), 30-year recovery of *P*. *vivax*-infected patients (30-y-RI, N = 30), and healthy individuals (HI, N = 20) (Table 1). There was no significant difference between gender of the past *P*. *vivax* infection groups (*p*> 0.5). However, there was significant difference for their age, although no significant between 5-y-RI and 12-y-RI groups (*p* = 0.795). Comparation of the age between 30-y-RI and 5-y-RI or 12-y-RI showed *p*< 0.01. A total of 210 *P*. *vivax* blood-stage proteins were expressed using the wheat germ cell-free method (WGCF). The proteins used in this study were separated into 228 fragments and grouped into 12 categories (Fig 2 and S1 Table)

**Table 1:** Characteristics of patient clinical serum samples.

Characteristics	Serum samples from				
	AVM	5-y-RI	12-y-RI	30-y-RI	HI
Total	20	37	30	30	20
Year of *P*. *vivax* infection	2007	2007	2000	1960s-1970s	-
Time interval from the last *P*. *vivax* episode (years)	0	5	12	30	-
Gender (M/F) ^a^	12/8	21/16	20/10	13/17	10/10
Age, mean ± SD (year) ^b^	35 ± 16	50 ± 22	49 ± 16	65 ±10	27 ± 3
Age, range (year)	17–58	7–85	19–78	45–83	23–33

^a^ No significant difference between the past *P*. *vivax* infection groups (*p*> 0.5).

^b^ No significant difference between 5-y-RI and 12-y-RI groups (*p* = 0.795). There is significant difference between 30-y-RI and 5-y-RI/ 12-y-RI groups (*p*< 0.01).

### Profiling humoral immune response to *P*. *vivax* infection using protein arrays

#### First screening

To identify the IgG responses and SEMs of *P*. *vivax* infection, we screened a mixture of 37 5-y-RI serum against 228 *P*. *vivax* protein fragments, as previously described [26,27]. Mixtures of 20 HI serum and PBS were used as negative and blank controls, respectively. The four slides used in this study were analyzed individually to avoid bias. The MFI values of each slide were ranked from low to high respectively, and cut-off value was defined as the mean fluorescence intensity (MFI) plus two standard deviations (SD) of the first 70% MFI values. Proteins with MFI values higher than the cut-off values were selected for analysis. The number of selected proteins in the samples varied from 10 to 19. A total of 60 (26.3%, 60/228) candidate protein fragments were identified and selected for a second screening (S1A Fig). It was observed that the seroprevalence of PvGAMA protein was relatively low, and PvMSP1-42 protein was higher with 5-y-RI serum, as the result, PvGAMA and PvMSP1-42 proteins were selected for the second screening as negative and positive controls, respectively.

#### Second screening

To confirm their reactivity, the 60 *P*. *vivax* protein fragments selected in the first screening were tested using a mixture of 5-y-RI and HI serum. The △MFI value of each protein was obtained from the mixed of 5-y-RI serum was subtracted from the MFI value of the mixed HI serum. Nine proteins showed higher △MFI values than other proteins (S1B Fig). The top nine proteins and two controls were selected for comprehensive screening to evaluate the persistence profile of the IgG antibodies.

#### Comprehensive screening

**Fig 3:**
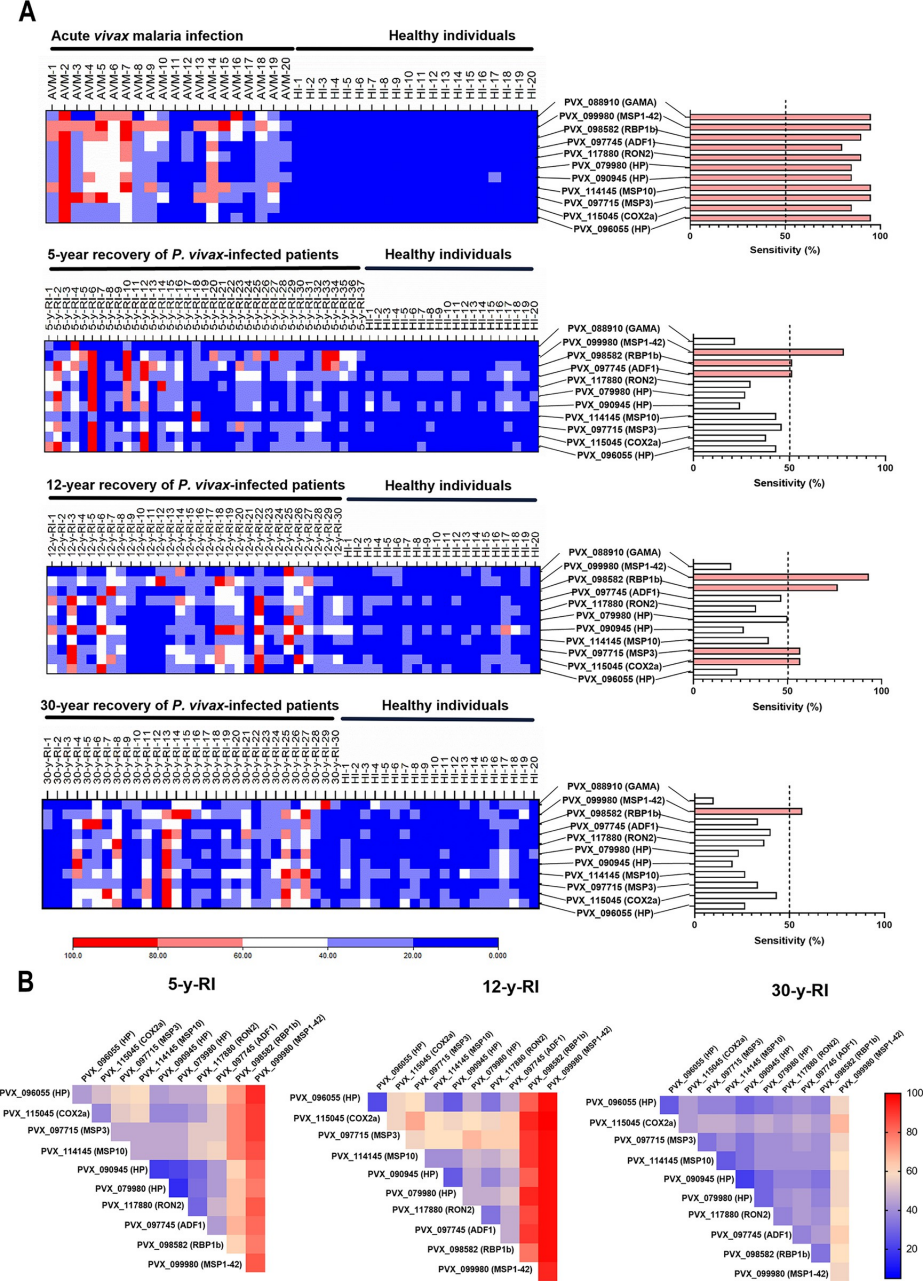
Immunoreactivity profiles of *P*. *vivax* proteins. (A) Reactions for the 11 proteins assayed with the sera from patients with acute *P*. *vivax* infection, from 5-year recovery of *P*. *vivax*-infected patients (5-y-RI), 12-year recovery of *P*. *vivax*-infected patients (12-y-RI) and 30-year recovery of *P*. *vivax*-infected patients (30-y-RI). The color bar 0–100 means the normalized MFI values of protein-serum reactions. (B) Proportion of positive reactions for paired proteins tested with sera from 5/12/30-y-RI. The color bar 0–100 means the sensitivity of two combined proteins reacting with the sera from 5/12/30-y-RI.

The cut-off value was defined as the mean plus 2SD of the MFIs with individual sera of HI. A response was considered positive if the MFI was above the cut-off. The proportion of positive reactions for the 11 tested proteins ranged from 100% to 16.2%. The proportions of sera from patients with acute *P*. *vivax* infection (AVM, N = 20) were all above 50% for 11 proteins, including proportions >95% for five proteins: PVX_099980 (MSP1-42), PVX_088910 (GAMA), PVX_114145 (MSP10), PVX_097715 (MSP3), and PVX_096055 (hypothetical protein, HP) (Fig 3A). The proportion of positive reactions >50% in the sera from 5-y-RI was detected for only three proteins: PvMSP1-42 (78.4%), PVX_098582 (RBP1b) (51.4%), and PVX_097745 (ADF1) (51.4%). PvGAMA accounted for the lowest proportion (21.6%). Using the sera from 12-y-RI, there were four proteins with >50% positive reactions: PvMSP1-42 (93.3%), PvRBP1b (76.7%), PvMSP3 (56.7%), and PVX_115045 (COX2a) (56.2%). For PvGAMA, the proportion was 20%. With sera from the 30-y-RI, only PvMSP1-42 showed >50% positive reactions (56.7%). For PvGAMA, the proportion of positive results was 10%. Overall, the comprehensive screening data showed that PvMSP1-42 had the highest proportion of positive reactions, and PvGAMA had the lowest, regardless of the exposed sera tested (S2 Fig and S2 Table).

Except for PvGAMA, the 10 selected proteins were paired to test the proportion of positive reactions with the sera from recovery of *P*. *vivax*-infected patients. The analysis showed that the proportion of positive reactions for the proteins increased when combined with PvMSP1-42. In the sera of 5-y-RI and 12-y-RI, PvRBP1b and PvMSP1-42 paired proteins showed a significantly higher proportion of positive reactions. In the 30-y-RI sera, only PvMSP1-42 showed a high proportion of positive reactions, indicating that PvMSP1-42 induces a long-term humoral immune response (Fig 3B).

### Physicochemical parameters and *in silico* humoral immune responses induced by PvMSP1-42 and PvGAMA

Except the signal peptide, full length of PvMSP1-42 and PvGAMA were used for the physicochemical and *silico* analysis. First, we found that the instability index ranged from 16.9 to 38.8, indicating that the PvMSP1-42 and PvGAMA were very stable [28]. Second, the solubilities of PvMSP1-42 and PvGAMA were estimated to be 0.603 and 0.518, by using the Protein-Sol server (https://protein-sol.manchester.ac.uk/), indicating that both proteins were highly soluble (both expected values were above the cut-off value of 0.45) [29]. Third, we found that the average antigenicity of PvMSP1-42 and PvGAMA were 0.731 and 0.7915, respectively, which were above the cut-off value (0.50) and indicated with strong immunogenicity [30] (S3 Table).

The C-ImmSim model describes the immune response of a mammalian system to antigens at the cellular level [31]. *In silico* analysis, it was found that PvMSP1-42 immunization produced 180,000/ml antibodies (IgM and IgG) and 82,000/ml antibodies (IgG1 and IgG2) on 43 days after immunization. By comparison, PvGAMA immunization produced 118,000/ml and 37,000/ml antibodies, respectively (S3A Fig). In addition, the results showed that the population per state of active B cell was 750 cells/mm^3^ and B memory cells population was 540 cells/mm^3^ in PvMSP1-42 immunization. For PvGAMA, the population per state of active B cells and B memory cells was 650 cells/mm^3^ and 490 cells/mm^3^, respectively (S3B and S3C Fig). Results of BCpreds, BepiPred2.0 and ABCpred server found that the predicted number of liner B-cell epitopes of PvMSP1-42 and PvGAMA did not vary significantly (S3D Fig).

### Antigenicity of PvMSP1-42 and PvGAMA

**Fig 4:**
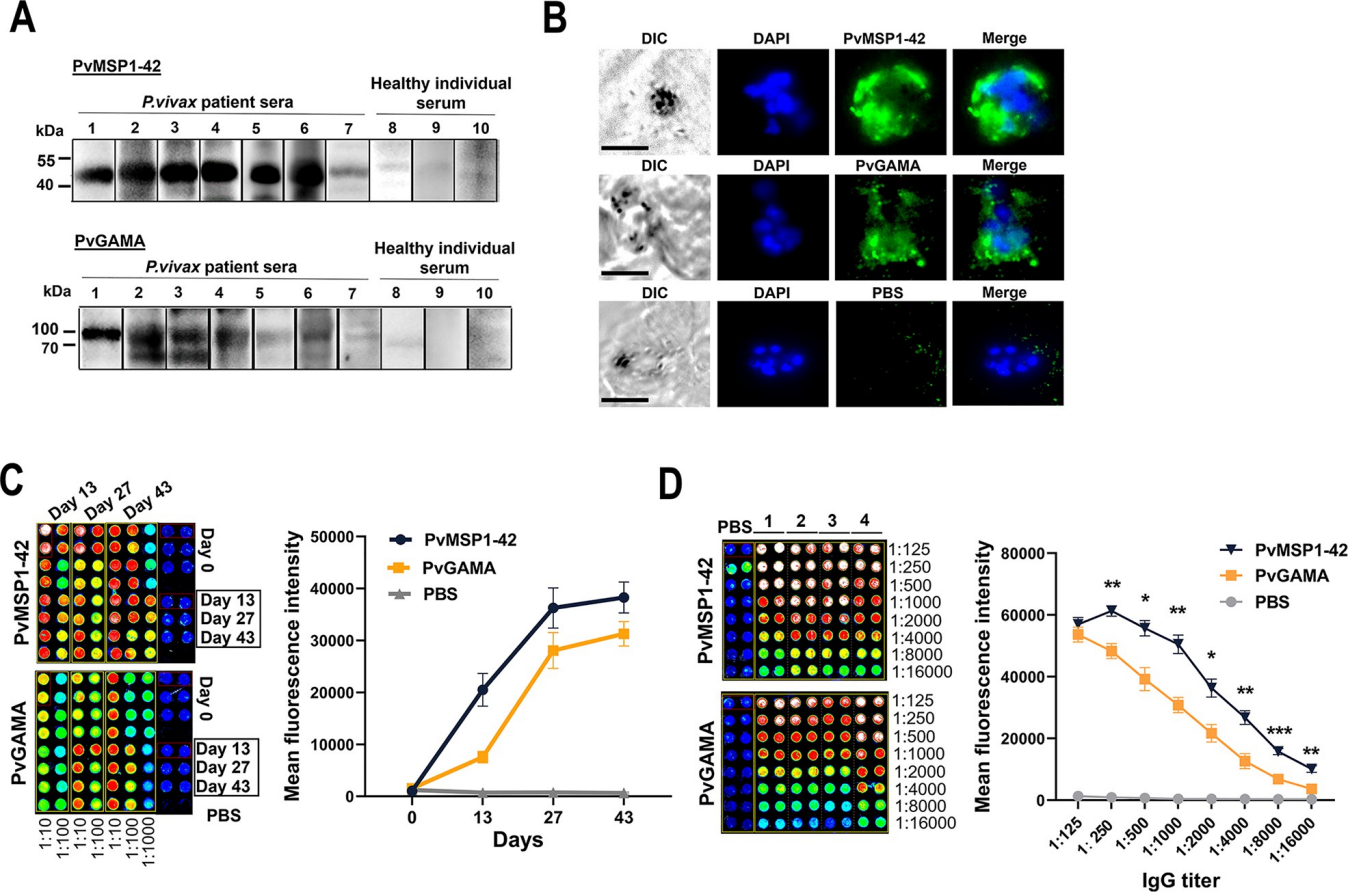
Humoral immune responses in PvMSP1-42 or PvGAMA-immunized mice. (A) Western blot analysis of PvMSP1-42 and PvGAMA probed with AVM serum (lanes 1–7) and healthy individual serum (lanes 8–10). (B) Subcellular localization of PvMSP1-42 and PvGAMA proteins in *P*. *vivax* positive blood smears. The nuclei were visualized using DAPI (blue). Bars represent 5 μm. (C) Antibody titers of sera obtained from PvMSP1-42-and PvGAMA or PBS immunized mice. Blood samples were collected on days 13, 27, and 43. (D) Series of two-fold dilutions (from 1:125 to 1:16,000) of sera collected on day-43 and assayed with PvMSP1-42 and PvGAMA proteins; the linearity and replicability of the data are shown in the graph. *p< 0.05, **p< 0.01, ***p< 0.001. Unpaired Student’s t-test. Error bars, mean ± SEM.

Consistent with the bioinformatic analysis, PvMSP1-42 comprises 380 amino acids with a calculated molecular mass of 43.2 kDa, and PvGAMA comprises 750 amino acids with 80.6 kDa were selected for expression (S4A Fig). We produced large amounts of recombinant PvMSP1-42 and PvGAMA using an *E*. *coli* system and purified them using a Ni-NTA affinity column. On SDS-PAGE and PVDF, the recombinant proteins revealed their molecular mass and a His-tag (S4B and S4C Fig). As *P*. *vivax* crude proteins were not available, we used *P*. *falciparum* crude proteins to indirectly validate that the serum of immunized mice with recombinant PvMSP1-42 or PvGAMA protein could bind to the natural *Plasmodium* protein. Western blot analysis of *P*. *falciparum* lysate probed with anti-PvMSP1-42 antisera confirmed that full-length MSP1 was proteolyzed to form 19, 42, 50, 80, and >200-kDa fragments as expected [32]. Blot probed with anti-PvGAMA antisera showed that GAMA underwent proteolytic processing to form 18-, 48-, and 80-kDa products as expected [33] (S4D Fig). We assayed the sera from AVM and HI to detect PvMSP1-42 and PvGAMA recombinant proteins and found that proteins expressed by *E*.*coli* could be detected in the serum of AVM, showing PvMSP1-42 protein bands of higher intensity than the PvGAMA protein bands (Fig 4A). We confirmed that the two proteins expressed in *E*. *coli* were immunogenic. Using positive *P*. *vivax* blood films, we observed that antibodies in sera from PvMSP1-42- and PvGAMA-immunized mice bound to *P*. *vivax-infected* red blood cells (Fig 4B).

After BALB/c mice were immunized subcutaneously with recombinant PvMSP1-42, PvGAMA or PBS emulsified with Freund’s adjuvant (S4E Fig), we measured the level of immune response (IgG titer) in immunized BALB/c mice against recombinant PvMSP1-42, PvGAMA protein, or PBS (negative control) using a protein array. After primary immunization, low or negligible titers of anti-PvMSP1-42 and anti-PvGAMA antibodies were detected. The IgG titers against PvMSP1-42 and PvGAMA proteins increased after the second immunization and reached high levels (MFIs for PvMSP1-42 and PvGAMA proteins at 179,541 and 197,965, respectively) 2 weeks after tertiary immunization (day 43 after immunization) (Fig 4C). The MFIs of PvMSP1-42 and PvGAMA were above 1,000 when the serum obtained after 43 days of immunization was diluted by 16,000. In addition, except for the highest concentration, the antibody titer of PvMSP1-42 was significantly higher than that of PvGAMA, preliminarily suggesting that PvMSP1-42 may have stronger immunogenicity (Fig 4D).

### PvMSP1-42 protein induced more antibody-secreting cells

**Fig 5:**
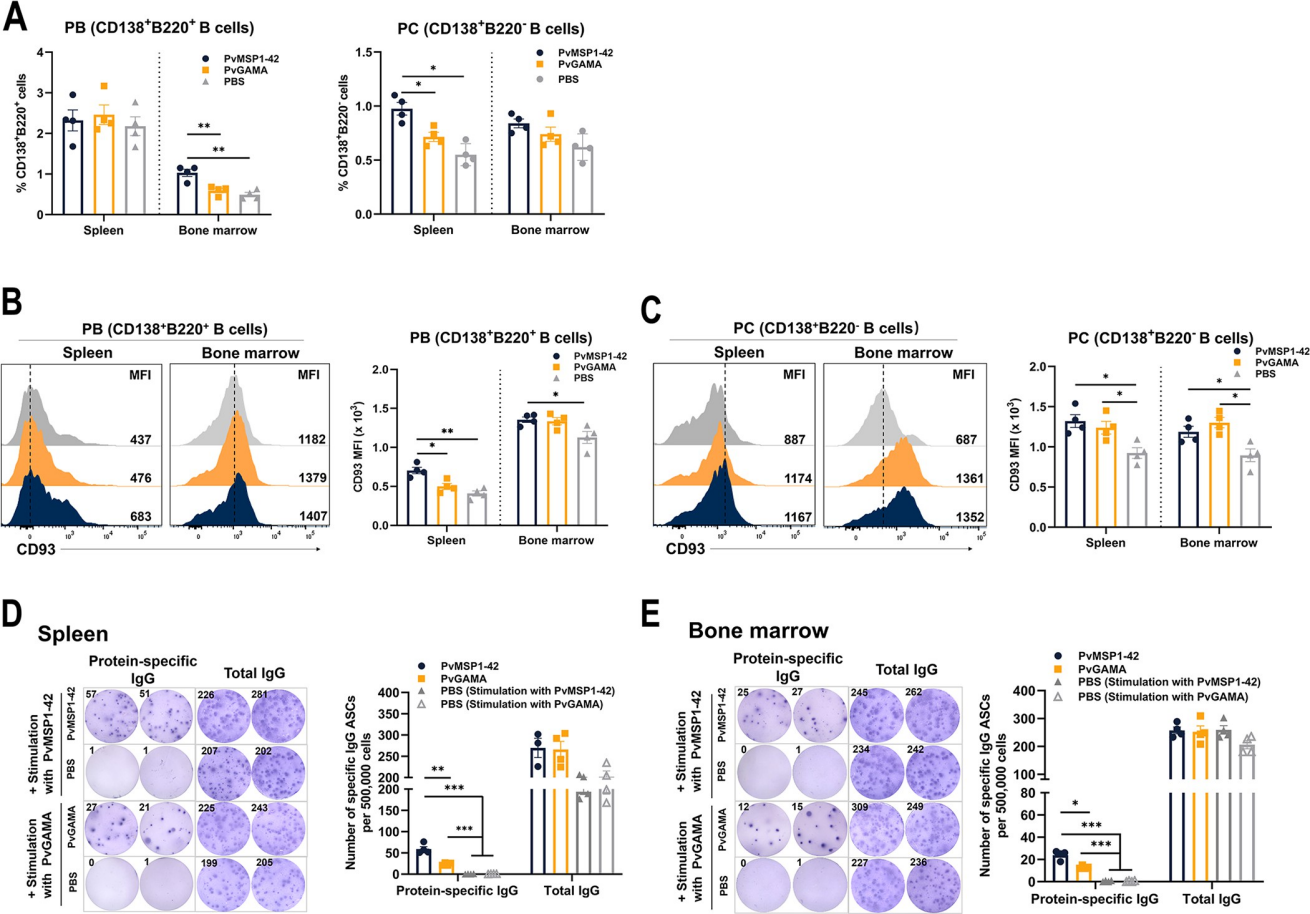
Number of ASCs in the spleen and bone marrow of immunized mice. (A). The diagrams present the frequency of plasmablasts (CD138^+^B220^+^) (on the left) and plasma cells (CD138^+^B220^−^) (right) detected by FACS in the spleen and bone marrow of PvMSP1-42-, PvGAMA-, and PBS-immunized mice. The graphs present histograms of CD93 expression in CD138^+^B220^+^ B cells (B) and CD138^+^B220^-^ B cells (C) detected by FACS in the spleen and bone marrow of PvMSP1-42-, PvGAMA-, and PBS-immunized mice. The graph on the right summarizes the data. MFI, mean fluorescence intensity. (D) Representative B cell ELISpot data (left) and the number of PvMSP1-42 or PvGAMA-specific IgG-producing ASCs and total IgG in total cell suspensions in the spleen (right) prepared from individual mice. (E) Representative B cell ELISpot data (left) and the number of PvMSP1-42 or PvGAMA-specific IgG-producing ASCs and total IgG in total cell suspensions in the bone marrow (right), which were prepared from individual mice. Results are expressed as the mean ± SEM (n = 4) from one representative experiment out of three with similar results. *p< 0.05, **p< 0.01, ***p< 0.001.

Next, we investigated the possible cause for the production of long-lived antibodies induced by PvMSP1-42 in a murine model (S5A Fig). We studied both antibody-secreting cells (ASCs): PBs, defined as CD138^+^B220^+^, which are short-lived, proliferate rapidly, and mainly produce low-affinity IgM antibodies, and PCs (plasma cells), defined as CD138^+^B220^-^, which are long-lived and secrete high-affinity antibodies (S7 Fig). We observed that the percentage of PBs in the bone marrow and PCs in the spleen of PvMSP1-42-immunized mice was higher than that in PvGAMA- or PBS-immunized mice on day 43 post-immunization (Figs 5A and S5B). CD93 is required to promote the mature phenotype of LLPCs and the production of high-affinity antibodies [34]. In mice, subpopulations of plasma cells with different lifespans can also be distinguished by CD93 [35]. We quantified CD93 expression in ASCs after immunization with PvMSP1-42 or PvGAMA. In the spleen, PBs showed a higher level of CD93 in PvMSP1-42 immunized mice than in PvGAMA-immunized mice (Fig 5B), whereas no significant difference in the expression of CD93 in PCs was detected in mice immunized with the two proteins (Fig 5C).

The proportion of PCs in the spleen and the proportion of PBs in the bone marrow were higher in PvMSP1-42-immunized mice than in PvGAMA- or PBS-immunized mice on day 43 post immunization. This finding prompted us to investigate whether the proportion of protein-specific ASCs differed between PvMSP1-42- or PvGAMA- and PBS-immunized mice. We found that the proportions of ASCs in the spleen (Fig 5D) and bone marrow (Fig 5E) of PvMSP1-42-immunized mice were higher than the proportions of ASCs in PvGAMA-immunized mice. These observations suggest that PvMSP1-42 induces the production of ASCs at higher levels in mice.

### Multiple exposures to antigens result in the generation of cells associated with the formation of B cell memory response

**Fig 6:**
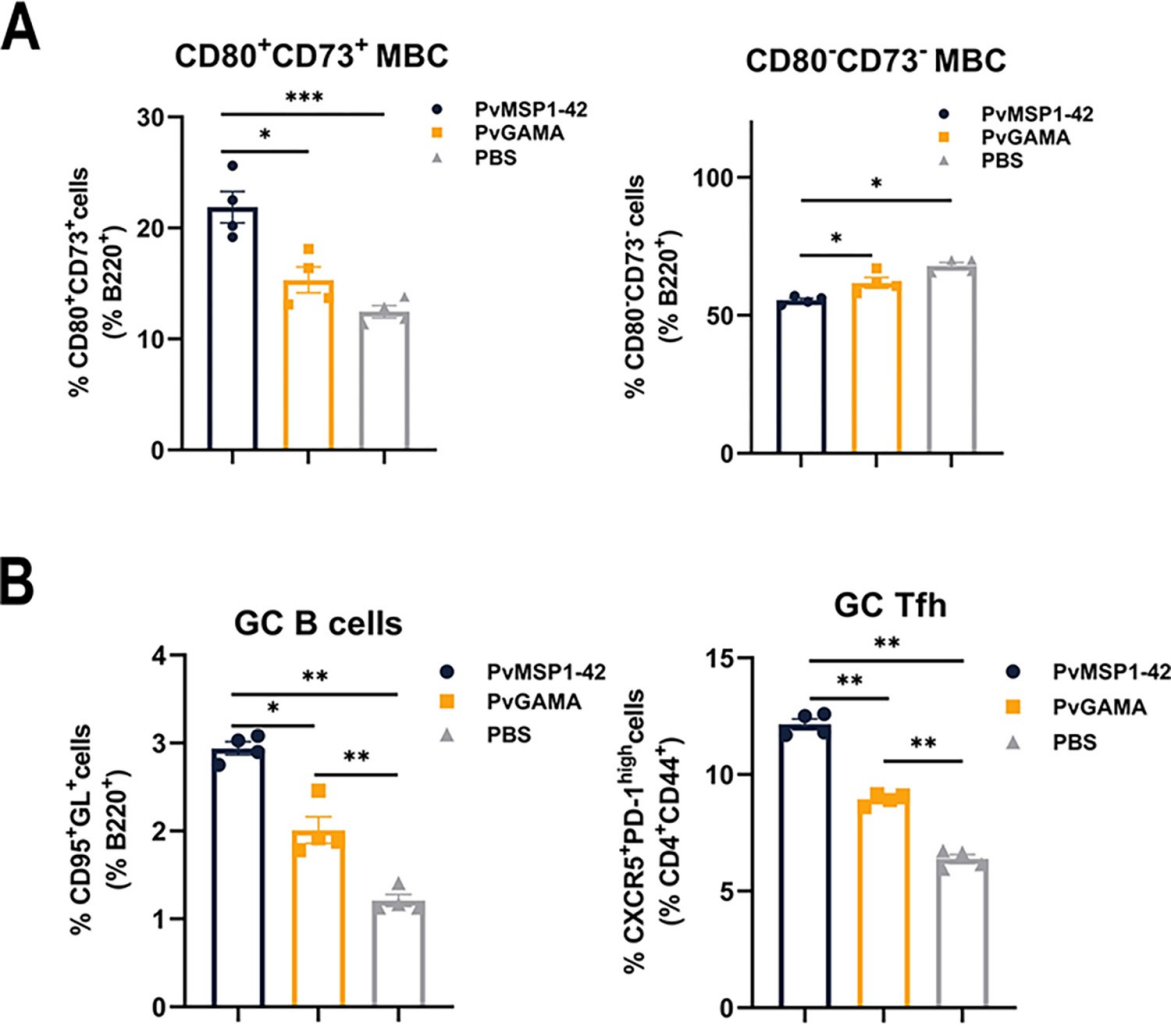
Frequency of cells related to the formation of the B cell memory response in PvMSP1-42- or PvGAMA-immunized mice. (A) Histograms present the frequency of CD73^+^CD80^+^ MBCs (left) and CD73^-^CD80^-^ naïve B cells (right) on B220^+^ pre-gated B cells of PvMSP1-42-, PvGAMA- and PBS-immunized mice. (B) Histograms present the frequency of splenic GC B cells (GL7^+^Fas^+^) on B220^+^ pre-gated B cells (left) and of GC Tfh cells (CXCR5^+^PD-1^high^) on CD4^+^ CD44^+^ pre-gated T cells (right) of PvMSP1-42-, PvGAMA- and PBS-immunized mice. Results are expressed as the mean ± SEM (n = 4) from one representative experiment out of three with similar results. *p< 0.05, **p< 0.01, ***p< 0.001.

Since MBCs can survive for a long time and rapidly differentiate into ASCs when re-exposed to an antigen and since high levels of anti-PvMSP1-42 antibodies were found in the sera of recovered *P*. *vivax*-infected patients, we investigated whether re-exposure to antigens induces germinal B cells to differentiate into early MBCs. Splenocytes from PvMSP1-42 immunized mice were stained for the two phenotypic markers associated with MBCs. The staining included proteins that regulate T-cell activity (CD80) and enzymes involved in the generation of adenosine from ATP (CD73) [36]. Phenotypic analysis revealed two subpopulations of MBCs: CD73^+^CD80^+^ and CD80^-^CD73^-^ cells (S7 Fig). The majority of the CD73^-^CD80^-^ B cells were IgD positive, indicating that these cells are primarily naïve B cells [37]. On day 43 post-immunization, we detected significantly more CD80^+^CD73^+^ MBCs and fewer CD73^-^CD80^-^ naïve B cells (Figs 6A and S6A) in the spleen of PvMSP1-42 immunized mice than in those of PvGAMA or PBS-immunized mice.

**Fig 7:**
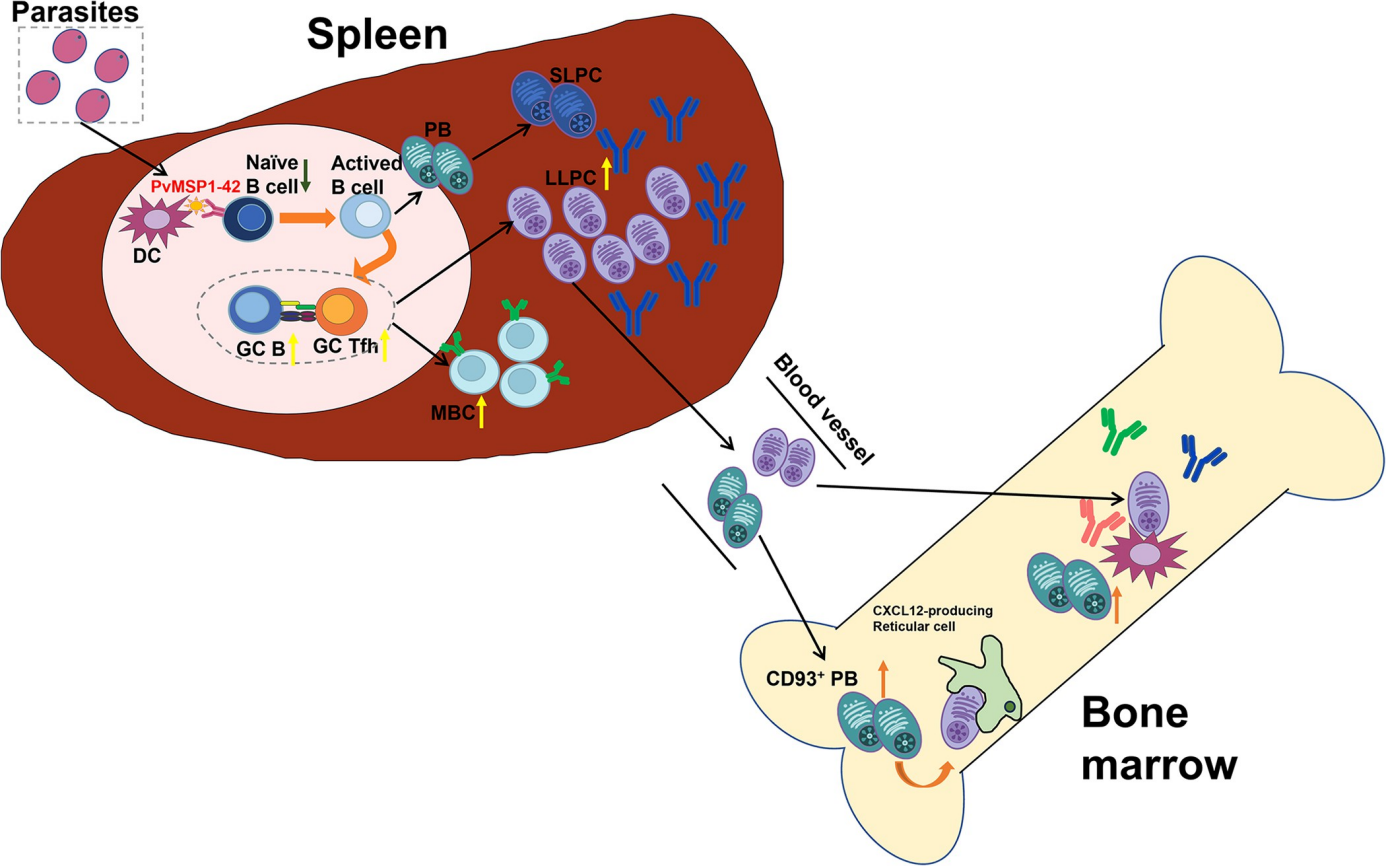
Schematic figure describing the changes in the number of cells associated with B cell memory formation after protein immunization. The quantity of both GC B cells and GC Tfh cells present within the spleens of PvMSP1-42-immunized mice were higher than those found within PvGAMA protein. In contrast, there were noticeably more CD80^+^CD73^+^ MBCs observed within the splenic tissue. Conversely, fewer CD73^−^CD80^−^ MBCs were detected in the spleen. It is worth mentioning that CD80^+^CD73^+^ IgG1 MBC subpopulations could generate PBs, which could give rise to a limited population size consisting primarily of GC B cells. Furthermore, an increased abundance pertaining to both plasma cell populations residing within splenic tissues as well as bone marrow compartments could be observed following administration with PvMSP1-42.

Next, we examined the frequency of GC B cells and Tfh cells in the spleen, as GC-dependent MBCs can undergo affinity maturation and class switching, enhancing the ability of these cells to mediate the clearance of certain pathogens [38]. On day 43 post-immunization, we observed that the number of B220^+^GL7^+^Fas^+^ GC B cells was higher in PvMSP1-42 immunized mice than in PvGAMA or PBS-immunized mice. As expected, PvMSP1-42 immunized mice developed a population of CXCR5^+^PD-1^hi^ GC Tfh cells [39, 40]. Again, the frequency of GC Tfh cells was higher in PvMSP1-42 immunized mice than in PvGAMA or PBS-immunized mice (Figs 6B, S6B and S6C). Taken together, these data show that the production of GC cells, GC Tfh cells, CD73^+^CD80^+^ MBCs, and PCs in the spleen and PBs in the bone marrow of PvMSP1-42 immunized mice was higher than that of PvGAMA-immunized mice (Fig 7).

## Discussion

*P*. *vivax* SEMs that reflect exposure to blood-stage *P*. *vivax* parasites, could play an important role in assessing progress towards malaria elimination [41]. Serological surveillance can be a useful tool for identifying areas of high transmission intensity or hidden asymptomatic reservoirs, especially as malaria transmission declines. The criteria for a serological marker are as follows: highly immunogenic, resulting in a robust and long-lasting serological response, and must be detectable after the initial acute infection has been cleared; highly specific, with minimal cross-reactivity with serum naïve individuals [42]. Over the past decade, *P*. *vivax* SEMs have been studied extensively. Recently, Longley *et al*. screened eight antigens from 342 *P*. *vivax* proteins using serum from longitudinal clinical cohorts of *P*. *vivax-*infected patients from Thailand, Brazil, and the Solomon Islands and identified candidate SEMs derived from individuals hypothesized carrying liver-stage hypnozoites [6]. They also showed low levels of cross-reactivity against the panel of eight *P*. *vivax* proteins in samples from human patients with *P*. *knowlesi* malaria [43].

In this study, we adopted a relatively unbiased approach for selecting a panel of SEMs by screening 210 *P*. *vivax* proteins and defining those that predicted *P*. *vivax* infection based on immunogenicity and antibody longevity. Due to the longtime span of the serum samples, there was a significant difference in the age of patients with documented past *P*. *vivax* infections. However, in this study, proteins that reacted with the serum of patients with documented past *P*. *vivax* infections (5-y-RI,12-y-RI and 30-y-RI) were considered as long-lived antibodies. Ten proteins were selected after three rounds of screening: PVX_096055 (HP), PVX_115045 (COX2a), PVX_097715 (MSP3), PVX_114145 (MSP10), PVX_090945 (HP), PVX_079980 (HP), PVX_117880 (RON2), PVX_097745 (ADF1), PVX_098582 (RBP1b), and PVX_099980 (MSP1-42). Both PvMSP1-42 [44–46] and PVX_088910 (GAMA) [47–49] have been reported to elicit a significant antibody response in the serum of *P*. *vivax* patients. Therefore, we focused our attention on these two proteins. PvGAMA which proved to be short-lived in our first screening, was included in our panel as a negative control. Merozoite membrane surface proteins (MSP3, MSP10, and MSP1-42) [42,50], RBP [51], RON2 [15], and GAMA [48], which were selected in our panel, were previously detected and proposed as SEMs [52]. Other *P*. *vivax* proteins selected by us have not yet been reported.

Of the 10 proteins selected, the seropositivity rate of sera from AVM patients was ≥ 80%; the lowest proportion was observed for PvADF1. However, the seropositivity rates of sera from the recovered *P*. *vivax-infected* individuals were lower. Positive serological reactions were detected in ≥50% of the three proteins (MSP1-42, RBP1b and ADF1) in the sera of 5-y-RI, for four proteins (MSP1-42, RBP1b, MSP3, and COX2a) in the sera of 12-y-RI, and only PvMSP1-42 in the sera of 30-y-RI. Finally, the most immunogenic and reactive protein was PvMSP1-42. Antibodies against PvMSP1-42 were detected in all sera, including those from AVM and recovered *P*. *vivax*-infected patients, although the seropositivity rates decreased over time from 95% in AVM to 78.4% in 5-y-RI, 93.3% in 12-y-RI, and 56.7% in 30-y-RI. MSP1 is expressed at merozoite stage and is involved in parasite erythrocyte invasion [53]. When *Plasmodium* invades red blood cells, the carboxyl fragment of 42 kDa is further processed into two immunogenic fragments of 33 and 19 kDa, that induce antibodies production and block the parasite invasion. In human and murine models, MSP1-42 has a highly conserved region recognized by B and T cells [15,54]. The MSP1-42 fragment increases IgG1, IgG2a, and IgG2b production (but not IgG3) in immunized mice and high titer T-cell-dependent antibodies have been produced by immunization with 19-kDa fragments [55]. Some studies in animal models have also shown that MSP1-19-specific IgG-producing MBCs and LLPCs can be detected up to 8 months after primary infection [56]. Besides PvMSP1-42, we also found that PvCOX2a had the highest seropositivity rate in the sera of 30-year recovery subjects (43.3%). Cytochrome c oxidase is an enzyme at the end of the mitochondrial electron transport chain in mammalian cells that transfers electrons to oxygen through proton gradient production, which is necessary for the production of the vast majority of ATP molecules in mammalian cells [57].

As PvMSP1-42 antibodies were detected in the serum of 30-year recovery subjects, we suspected that this was likely related to long-lasting humoral immunity. For the limitation of *P*. *vivax* crude protein obtained, *P*. *falciparum* crude protein was used in the process of detecting the immunogenicity of protein expression in *E*. *coli*. The homology of PvMSP1-42 and PfMSP1-42 was 40.62%, and the homology of PvGAMA and PfGAMA was 52.26%, compared the amino acid sequences by Basic Local Alignment Search Tool (BLAST) [29]. We next investigated whether PvMSP1-42-induced humoral and memory B cell response generates long-lived antibodies in a murine protein immune model. In areas with high *P*. *vivax* transmission, *Plasmodium*-specific LLPC and MBC cannot be effectively induced, and immune responses against *Plasmodium* blood-stage parasites are rarely obtained, even after repeated infections [58, 59]. However, in areas of relatively low transmission intensity, it has been shown that infected individuals can have durable immunity against *P*. *vivax* or *P*. *falciparum* [46, 60]. In our study, the confirmation of the absence of malaria infection was based on data from the malaria case reporting system and consultations with patients. In 2011, the last malaria case reported in Jiangsu Province, sera from the past *P*. *vivax* infections were collected in 2012, then they lived in malaria free area at least 1 year. The possibility of sterilizing immunity was uncertain for the ability of prevent infection of the long-lived antibodies were not confirmed in this study.

Several mechanisms have been proposed to explain for the transient nature of *Plasmodium*-specific humoral immunity, including expansion of PBs [61], CXCR3^+^ Tfh cells [62], regulatory T cells [63,64], and atypical MBCs [65,66]. However, for the maintenance of long-lived antibodies, it is generally agreed from extensive studies of humoral responses to immunization and acute viral infection, that two types of long-lived pathogen-specific B-cell lineage cells remain in the memory pool: LLPCs, which secrete specific antibodies for prolonged or even lifelong periods, and MBCs, which provide rapid and enhanced responses to secondary pathogen challenges. GC Tfh cell and GC B cell responses are critical for the generation of isotype-switched LLPCs and MBCs [67,68]. Here, using a murine protein immune model, we provide humoral and memory B cell response after the induction of PvMSP1-42. CD93, which is important for the maintenance of PCs in the bone marrow niche [69], is expressed on PBs and PCs, including LLPCs, which exhibit reduced cell cycle activity and high levels of isotropic immunoglobulin secretion and transcriptional network modification [70]. Previous reports have shown that a rapid decline in plasma cell responses in the spleen is accompanied by an increase in PCs in the bone marrow, which can rapidly differentiate into ASCs in secondary immune responses. Although we did not find a significant difference in CD93^+^ PCs in the spleen and bone marrow of immunized mice, we observed that the frequency of CD93^+^ PCs was significantly higher than that in PBS-immunized mice (*p*< 0.01). In fact, PvMSP1-42-specific ASCs were more abundant than PvGAMA-specific ASCs. CD93 was low in PBs in the spleen of PvMSP1-42 immunized mice. As there were more PBs in PvMSP1-42 immunized mice in the bone marrow, this suggests that once PBs are produced, they may migrate into the bloodstream and reach a survival niche (mainly in the bone marrow) where they receive survival signals and become LLPCs [71]. However, without further investigation of PCs and PBs in the bone marrow, we did not determine whether PBs in the bone marrow of PvMSP1-42 immunized mice differentiated into LLPCs. CD80, PD-L2, and CD73 are surface proteins expressed by MBCs that are used to classify MBCs into different phenotypes. CD73, a marker of GC-derived IgM^+^ MBCs, is expressed at low levels in GC-independent MBCs [72,73] and increases during the development of GC B cells [73,74]. We observed that the population of CD80^+^CD73^+^ MBCs in the spleen of PvMSP1-42 immunized mice increased, while naïve B cells decreased, and CD80^+^CD73^-^ and CD80^-^CD73^+^ MBCs populations, belonging to GC-independent MBCs, were present in low proportions. As previously reported [75,76], after three rounds of immunization with PvMSP1-42, we observed that the number of GC B cells and GC Tfh cells in the spleens of mice increased rapidly, which could lead to an increase in MBCs and LLPCs differentiated from GC B cells. Whether the results obtained from *in silico* or *in vivo*, the levels of PvMSP1-42 IgG antibody are consistently higher than those of PvGAMA. The populations of active B cells and B memory cells in PvMSP1-42 immunization were higher than those in PvGAMA immunization. The results of *in silico* were consistent with *in vivo*.

Although our study has some limitations, such as the limited number of patient clinical serum samples tested and the absence of long-term follow-up of MBCs and ASCs in immunized mice, we clearly demonstrated that PvMSP1-42 induces long-lived anti-PvMSP1-42 antibodies in serum of *P*. *vivax* patients and more CD73^+^CD80^+^ MBCs, allowing IgG anti-PvMSP1-42 antibodies to be maintained for a long time in a murine protein immune model. A study with a larger sample size and improved surveillance should confirm the findings of long-term immunity induced by PvMSP1-42 antigen.

## Materials and methods

### Ethics statement

Blood samples were collected after approval of the protocols reviewed by the Institutional Review Board (IRB00004221) of the Jiangsu Institute of Parasitic Disease, Wuxi, China. The informed written consent and/or assent of all participants were obtained, the participants included children, who’s written consent was obtained from their parent.

The mice used in this study were obtained from the Experimental Animal Center of Yangzhou University (Yangzhou, China). All animal procedures were performed in accordance with protocols approved by the Laboratory Animal Ethics Committee of Yangzhou University (protocol number 202305004).

### Serum sample information

Samples were collected from three groups of individuals: patients with acute *P*. *vivax* malaria, recovered *P*. *vivax*-infected patients, and healthy individuals who had never been infected with *P*. *vivax*. Data on acute *P*. *vivax* malaria infection were collected from patients with mild symptoms and microscopically positive *P*. *vivax* parasitemia, at local health centers and clinics in Bengbu City, Anhui Province. The recovery samples were collected from Suqian City and Yancheng City, Jiangsu Province. The Healthy were collected from Wuxi City, Jiangsu Province. The sera were aliquoted and stored at -80°C.

Data from recovered *P*. *vivax*-infected patients were collected in 2012 and assigned to one of the three groups according to their history of malaria infection: 5-year (5-y-RI), 12-year (12-y-RI), and 30-year (30-y-RI) recovered individuals. Patients were defined as ‘5-year recovery individuals’ if they were infected with malaria in 2007 and had no history of malaria between 2007 and 2012. The definition of ‘12-year recovery individuals’ had similar restrictions. In contrast, ‘30-year recovery individuals’ were defined broadly as individuals with documented *P*. *vivax* malaria episodes in the 1960-1970s and no history of *P*. *vivax* malaria infection. Serum samples from healthy malaria-naïve individuals living in non-endemic areas of China were used as controls.

### Immunization of mice with recombinant proteins

Groups of four 6–8-week-old female BALB/c mice were immunized subcutaneously with approximately 50 μg of the recombinant protein or phosphate-buffered saline (PBS) emulsified with complete Freund’s adjuvant (Sigma-Aldrich, St. Louis, MO, USA). On days 14 and 28, the mice were challenged with either antigens or PBS emulsified with the same amount of incomplete Freund’s adjuvant (Sigma-Aldrich). Blood samples were collected the day before each injection. The mice immunized with PvMSP1-42 or PvGAMA were used as a murine protein immune model.

### Recombinant proteins

All target proteins used for immuno-screening by protein arrays were expressed using a WGCF system (CellFree Sciences, Matsuyama, Japan) as described previously [26,77]. The expression level and solubility of each protein were evaluated by western blot analysis, as described previously. In addition, two targets, PvMSP1-42 and PvGAMA, were expressed in *the E*. *coli* system for mechanistic studies. Briefly, fragments were amplified from the plasmid used for the WGCF expression system and cloned into the pET28a vector. The clones were transformed into *E*. *coli* Rosetta (DE3) strains for protein expression and purified using nickel-nitrilotriacetic acid (Ni-NTA). Purified proteins were confirmed by SDS-PAGE and Western blot analyses and quantified by BCA Protein Assay Kit (Thermo Fisher, Germany). Endotoxin content was also tested using the LAL Endotoxin Assay Kit (GenScript, Nanjing, China).

### Bioinformatic predictions

The immunogenicity and antigenicity of the proteins were predicted using VaxiJen v2.0 (https://www.ddgpharmfac.net/vaxijen/VaxiJen/VaxiJen) and ANTIGENpro server (https://scratch.proteomics.ics.uci.edu/). The theoretical isoelectric point (pI), solubility and instability index of the two proteins were predicted using the ExPASy ProtParam server. The protein’s pI informs the selection of an appropriate purification method for the protein. The instability index indicates the protein’s stability *in vitro*. If the predicted value is below the threshold of 40, it is considered to be stable, while an instability index range of 16.90 to 38.78 suggests a high level of protein stability [28]. Additionally, the Protein-Sol server (https://protein-sol.manchester.ac.uk/) was used to forecast how soluble the protein will be. A protein may have good solubility if the expected value is greater than the cut-off of 0.45 [29]. Immune stimulation was performed using the C-ImmSim server (https://150.146.2.1/C-IMMSIM/index.php), which can predict changes in B cell expression following protein stimulation [78]. Parameters were set to random seeds, simulation volume was set to the default value, and HLA was set to the server-recommended allele. The number of simulation steps was set to 264 and three injections of two different proteins were performed. The injection time was set to days 0, 14, and 28.

The three prediction algorithms, including BCpreds (https://ailabprojects2.ist.psu.edu/bcpred/predict.html), BepiPred2.0 (https://services.healthtech.dtu.dk/services/BepiPred-2.0/) and ABCpred (https://webs.iiitd.edu.in/raghava/abcpred/index.html) were used to predict linear B-cell epitopes of PvMSP1-42 and PvGAMA. Threshold values were defined as 0.50, 0.51, and 75% for BepiPred, ABCpred, and Bcpreds, respectively. Fragments with more than nine amino acids and predicted for at least two of prediction algorithms were considered as a linear B-cell epitope [79,80].

### Isolation of mouse spleen and bone marrow cells

There is a positive correlation between protein-specific MBCs and antibody levels [81,82]. Therefore, we examined immune memory-related cells at day 43 since antibody levels were highest at this time. Spleen and bone marrow cells isolated on day 43 after immunization were stimulated using established protocols [83]. Fresh whole mouse spleens were minced into small pieces using sterile scissors in Petri dishes containing 10 ml of pre-cooled RPMI 1640 (Solarbio, Beijing, China). Spleen tissue pieces were gently crushed through a 200-mesh filter using a syringe plunger until the remaining spleen tissue became white. The tibia and femur muscles of the mice were separated using sterile surgical scissors and transplanted into sterile Petri dishes containing RPMI 1640. The ends of the mouse femurs and tibias were cut off, and the bone marrow cavity was rinsed repeatedly with a 1 ml syringe and filtered through a 200-mesh filter into a centrifuge tube. The cells were then centrifuged at 559 × g for 5 minutes, and the supernatant was discarded. The pellet was resuspended in red blood cell lysis buffer and incubated for 5 minutes at room temperature. After two washes with RPMI 1640, the cells were added to complete the RPMI medium, counted, and placed on ice for subsequent use.

### SDS-PAGE and Western blot

To determine protein expression levels and immunogenicity, recombinant proteins and *P*. *falciparum* parasites rich in schizonts (parasitemia> 1%) extracted in SDS-PAGE loading buffer were separated by 12.5% SDS-PAGE, transferred to an Immobilon PVDF membrane (Millipore, Burlington, MA), then detected with His-tag antibodies (1:5,000, Abmart, Shanghai, China) and PvMSP1-42/PvGAMA/PBS immunized sera (1:4, 000), respectively. And then membranes were incubated with secondary HRP-conjugated goat anti-mouse IgG (1:10,000, ThermoFisher Scientific, Rockford, IL, USA) according to previously described protocols [84]. Blots were developed by enhanced chemiluminescence (ECL, Beyotime) using Tanon 1600 (Tanon, Shanghai, China).

### Fluorescence microscopy

As previously described [33], fluorescence microscopy was performed after the *P*. *vivax*-infected blood smears were fixed with ice acetone. Mouse anti-PvMSP1-42 (1:10), mouse anti-PvGAMA (1:10), or mouse anti-PBS (1:200) sera were used as primary antibodies. The secondary antibody Alexa-488 goat anti-mouse IgG (1:500, Invitrogen) and nuclear stain 4’, 6’-diamidino-2-phenylindole (DAPI, 1:1,000, Invitrogen) were used for all treatments. Slides were mounted in Prolong Gold antifade reagent (Invitrogen) and visualized under oil immersion using a fluorescence microscope (Carl Zeiss MicroImaging, Thornwood, NY, USA). Images were acquired using the Zen software (Carl Zeiss MicroImaging).

### Protein arrays

Protein arrays for antigen screening were performed on nickel-chelate surface slides. The method was similar to that described previously [85]. Briefly, crude protein was added to each well and incubated for 1 hour at 37°C after blocking with 5% BSA in PBS-Tween (0.1%) (PBST). The chips were then tested with sera from *P*. *vivax* patients, recovered individuals, and healthy individuals (1:10) that had been pre-absorbed against wheat germ lysate (1:100) to block anti-wheat germ antibodies. Alexa Fluor 546 goat anti-human IgG (1:200, Invitrogen) in PBS was used as the detection antibody, and fluorescence signals were detected using a fluorescence scanner (ScanArray Express, PerkinElmer, Boston, MA, USA) and quantified as previously described [26]. To normalize the antibody array data, the fluorescence intensities were divided by the cut-off value. After preliminary screening, the humoral immune response of malaria patients was further evaluated using proteins with high immunoreactivity. Finally, the serum from all individuals was used for secondary screening to identify the most reactive protein.

We also applied adhesive microscope slides (Citotest Scientific, Jiangsu, China) to the protein array to detect the immunoreactivity of the proteins expressed by the *E*. *coli* expression system and antibody titers after immunization of mice. Purified proteins, PvMSP1-42 and PvGAMA (1 μl/spot, 20 ng/μl), were spotted into duplicate wells of the arrays and incubated for 2 hours at 37°C. After blocking with 1 μl of 5% BSA in PBST for 1 hour at 37°C, the chips were probed with serum for 1 hour at 37°C. Sera from patients with acute *P*. *vivax* infection and healthy individuals were used to detect immunoreactivity for the two proteins, and protein immune mouse sera at dilutions of 1:250, 1:500, 1:1,000, 1:2,000, 1:4,000, 1:8,000, 1:16,000, and 1:32,000 were used to detect antibody titers. Antibodies were visualized using Alexa Fluor 555 donkey anti-mouse IgG (1:100, Beyotime) in PBS and scanned using a LuxScan 10 K-B microarray scanner (CapitalBio Technology, Beijing, China).

### Flow cytometry

Spleen and bone marrow cells were stained with Biolegend antibodies: CD80 (clone 16-10A1), CD73 (clone TY/11.8), CD95 (clone SA367H8), CD3 (clone 17A2), CD4 (clone GK1.5), CD44 (clone IM7), CXCR5 (clone L138D7), and PD-1 (clone 29F.1A12), or BD Bioscience antibodies: B220 (clone RA3-6B2), T and B cell activation antigen (clone GL7), CD138 (clone 281–2), CD93 (clone AA4.1) washed, and analyzed on a FACSCalibur flow cytometer (BD Biosciences). Data were analyzed using FlowJo v10 software (TreeStar, La Jolla). Profiles are presented as 5% probability contours with outliers.

### In vitro B-cell restimulation and ELISpot assays

Red blood cells-depleted splenocytes and bone marrow cells were cultured at 5×10^5^ cells/ml in RPMI 1640 medium, 2 mM L-glutamine, 100 U/ml penicillin, and 0.1 mg/ml streptomycin supplemented with 10% fetal bovine serum (FBS, all from Solarbio) and 50 μM β-mercaptoethanol (Sigma) at 37°C for 3 days in the presence or absence of 25 μg/ml of PvMSP1-42 and PvGAMA protein.

PvMSP1-42 and PvGAMA-specific bone marrow or spleen ASCs were determined after 3 days of antigen stimulation by ELISPOT assay as previously described [86]. The polyvinylidene fluoride 96-well plates were coated with PvMSP1-42 or PvGAMA protein at 10 μg/ml overnight at 4°C, and then cultured with splenocytes or bone marrow cells at 5×10^5^ cells/ml in RPMI medium containing 50 μM β-ME and 10% FBS for 20 hours. After thorough washing with PBST, diluted biotin-labeled goat anti-mouse IgG (H+L) (1:2,000, Beyotime) and AP-labeled streptavidin (1:2,000, Beyotime) were added, followed by the addition of the BCIP/NBT substrate (Sigma) for ELISPOT (Mabtech). The filtered plates were rinsed with deionized water when clear spots appeared. ASCs were expressed as spot-forming cells (SFCs) in the wells, and the spots were counted using the ImmunoSpot Single-Color ELISPOT Enzymatic software of the S6 Entry M2 ELISPOT reader (C.T.L., Cleveland, USA)

### Statistical analysis

GraphPad Prism (version 8.0; GraphPad, San Diego, CA, USA) statistical analysis program was used to perform a two-tailed Student’s t-test on unpaired samples for comparisons between experimental groups and one-way ANOVA was used in three groups comparison. For all tests, a *p*-value was considered significant if it was less than < 0.05 (*), < 0.01 (**), and < 0.001 (***).

## Supporting information

S1 Fig Immunoreactivity profiles of *P*. *vivax* proteins in the first and second screenings. (A) In the first screening pooled mixed sera from 5-year recovery individuals (5y-RI) and 228 *P*. *vivax* protein were used. The first screening results of protein chip (on the left) and statistical analysis (on the right). AP, assuming that is positive; AN, assuming that is negative. Numbers correspond to the chip used: 1 for chip 1, 2 for chip 2, 3 for chip 3 and 4 for chip 4. (B) For the second screening,60 proteins selected from the first screening were reacted with the mixed sera from 5y-RI and healthy individuals (HI). The results of protein chip (on the left) and statistical analysis (on the right). The red circles were indicated the target proteins selected for comprehensive screening. △MFI = MFI (5y-RI)–MFI (HI). C, PvMSP1-42 and PvGAMA were used as positive and negative control, respectively. Numbers correspond to the chip used: 1 for chip 1; 2 for chip 2.
(TIF)

S2 Fig Evaluation of IgG antibody persistence against the 11 proteins in sera from 5y-RI, 12y-RI and 30y-RI. (TIF)

S3 Fig Immune stimulation of innate immune cells prediction. (A) The simultaneous display of both relative antibody responses and antigen concentration, along with the presence of IgG, serves as evidence of immunogenicity of the two proteins. (B) B cell population (cells/mm^3^) and (C) B cell population per state (cells/mm^3^), these graphs described the relative number of plasma cells that produce antibodies. The value of IgM + IgG, IgG1 + IgG2, B memory cells and active B cells for day 43 was shown in red. (D) *In silico* prediction of linear epitopes was performed on the PvMSP1-42 and PvGAMA sequence using Bcpreds, Bepipred, and ABCpred algorithms. Green boxes indicated the predicted liner B-cell epitopes. Sequences longer than nine amino acids and predicted by at least two algorithms were considered as B-cell linear epitopes.
(TIF)

S4 Fig Humoral immune responses in PvMSP1-42- or PvGAMA-immunized mice. (A) Diagram of PvMSP1-42 (aa 1350–1729) and PvGAMA (aa 22–771) fragment used for recombinant protein expression. (B) Electrophoresis gels presenting the expected bands of the *pvmsp1-42* (1140 bp) and *pvgama* (2254 bp) genes. (C) SDS-PAGE and western blot confirming the presence of PvMSP1-42 (~ 42 kDa) and PvGAMA (~ 80 kDa) (M: marker). (D) Western blot confirming the presence of PvMSP1-42 and PvGAMA in *P*. *falciparum* lysate were detected in the serum of immunized mice. Arrows represent specific bands for each recombinant protein. (E) Design of the strategy used to immunize mice.
(TIF)

S5 Fig Number of ASCs in the spleen and bone marrow of immunized mice. (A) The flow chart presents the strategy used to detect B cell memory formation-related cells after protein immunization in mice. (B) Representative dot plots of PBs (CD138^+^B220^+^) and PCs (CD138^+^B220^-^) detected by FACS in the spleen and the bone marrow of PvMSP1-42-, PvGAMA- and PBS-immunized mice.
(TIF)

S6 Fig Frequency of cells related to the formation of B cell memory response in PvMSP1-42- or PvGAMA-immunized mice in spleen. (A) Representative dot plots of CD73^+^CD80^+^ MBCs and CD73^-^CD80^-^ naïve B cells on B220^+^ pre-gated B cells in sera from PvMSP1-42-, PvGAMA- and PBS-immunized mice. (B) Representative dot plots of splenic GC B cells (GL7^+^Fas^+^) on B220^+^ pre-gated B cells in sera from PvMSP1-42-, PvGAMA- and PBS-immunized mice. (C) Representative dot plots of GC Tfh cells (CXCR5^+^PD-1^high^) on CD4^+^ CD44^+^ pre-gated T cells in sera from PvMSP1-42-, PvGAMA- and PBS-immunized mice.
(TIF)

S7 Fig Gating strategy for cell phenotyping. Lymphocytes were identified by forward scatter (FSC-H) vs side scatter (SSC-H) density plots. Using FSC-H and FSC-A to identify single cells, B cells were defined as B220^+^, memory B cells subsets of naïve and mature B cells were defined by the relative expression of CD73 and CD80 for naïve B cells (CD73^-^CD80^-^) and CD73^+^CD80^+^ memory B cell subsets, plasma cells were defined as B220^-^CD138^+^ and plasmablasts as B220^+^CD138^+^. Germinal center B cells were defined as B220^+^Fas^+^GL-7^+^, Germinal center T follicular helper (GC Tfh) cells as CD3^+^ CD4^+^ CD44^+^ CXCR5^+^ PD-1^hi^ and T follicular helper (Tfh) cells as CD3^+^ CD4^+^ CD44^+^ CXCR5^+^ PD-1^int^.
(TIF)

S1 Table Classification of putative antigenic proteins on predicted subcellular function or location. (PDF)

S2 Table Comprehensive screening of the 11 *P*. *vivax* proteins with different malaria historic individual sera /Prevalence of IgG antibodies against the 11 *P*. *vivax* proteins. (PDF)

S3 Table Physicochemical parameters and antigenicity prediction of PvMSP1-42 and PvGAMA proteins. (PDF)

S1 File The protocol for flow cytometry and ELISpot. (DOCX)

S1 Data The raw data of Table 1, Figs 1, 2, 3, 4, 5, 6, S1, S2 and S4. (XLSX)

S2 Data The raw data of Fig 4B in this study. (ZIP)
