# Supplementary figures and images for: Plasmodium vivax serological exposure markers: PvMSP1-42-induced humoral and memory B-cell response generates long-lived antibodies
Source: PLoS Pathog. 2024 Jun 28;20(6):e1012334. doi: 10.1371/journal.ppat.1012334 (PMC11239109; doi:10.1371/journal.ppat.1012334)

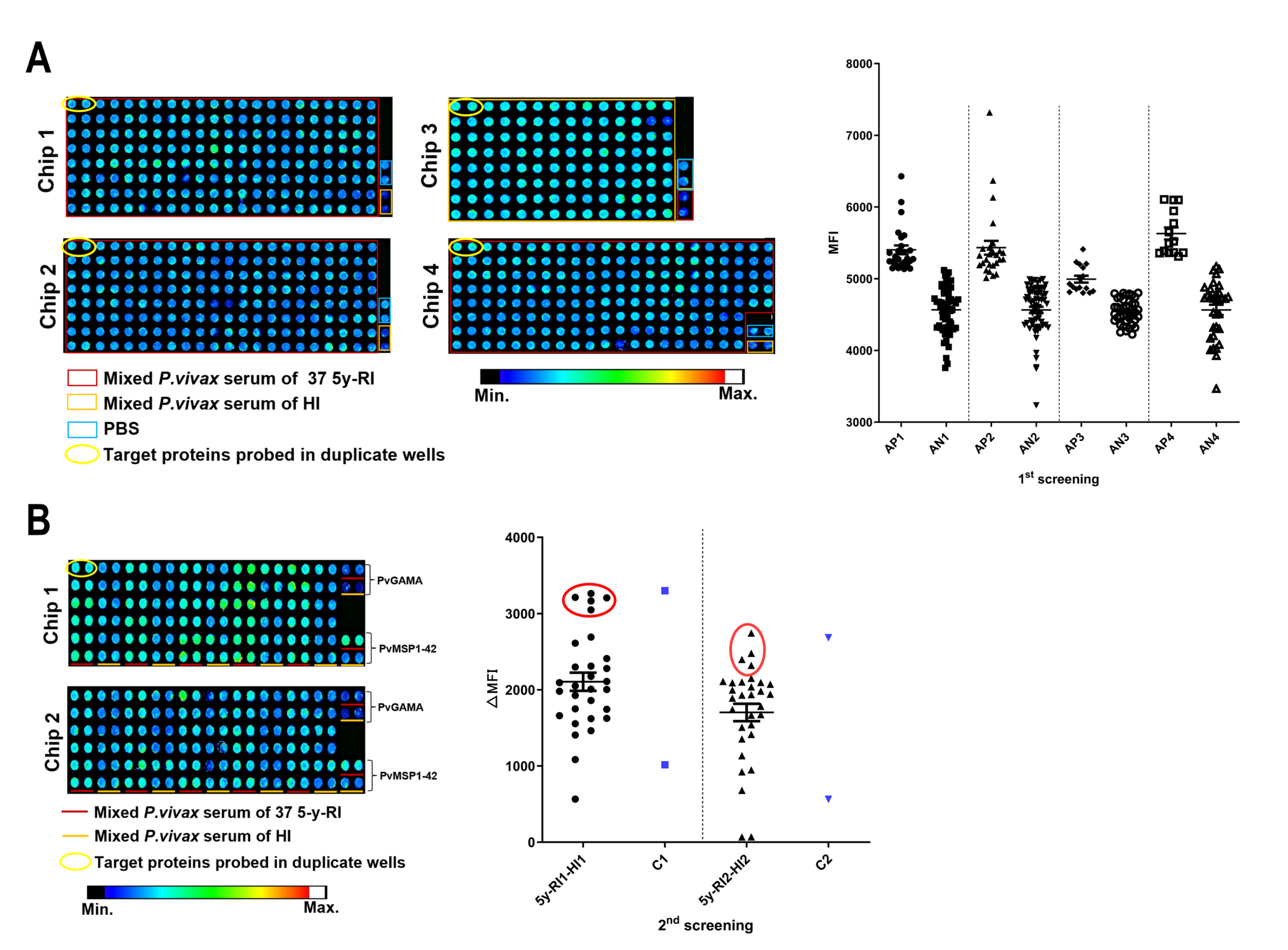

Supplement: S1 Fig — (A) In the first screening pooled mixed sera from 5-year recovery individuals (5y-RI) and 228 P. vivax protein were used. The first screening results of protein chip (on the left) and statistical analysis (on the right). AP, assuming that is positive; AN, assuming that is negative. Numbers correspond to the chip used: 1 for chip 1, 2 for chip 2, 3 for chip 3 and 4 for chip 4. (B) For the second screening,60 proteins selected from the first screening were reacted with the mixed sera from 5y-RI and healthy individuals (HI). The results of protein chip (on the left) and statistical analysis (on the right). The red circles were indicated the target proteins selected for comprehensive screening. △MFI = MFI (5y-RI)–MFI (HI). C, PvMSP1-42 and PvGAMA were used as positive and negative control, respectively. Numbers correspond to the chip used: 1 for chip 1; 2 for chip 2. (TIF) [file ppat.1012334.s001.tif]

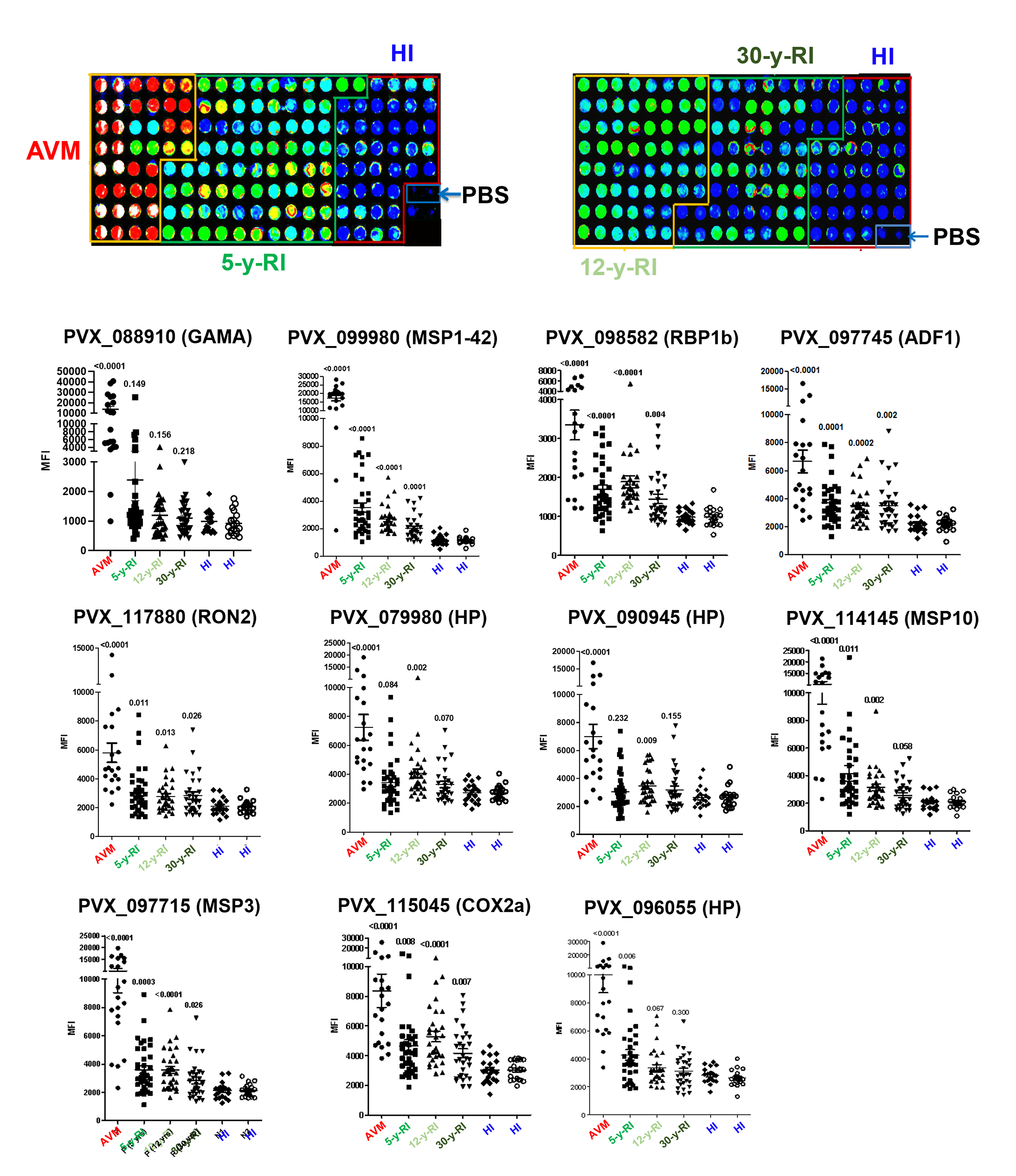

Supplement: S2 Fig — (TIF) [file ppat.1012334.s002.tif]

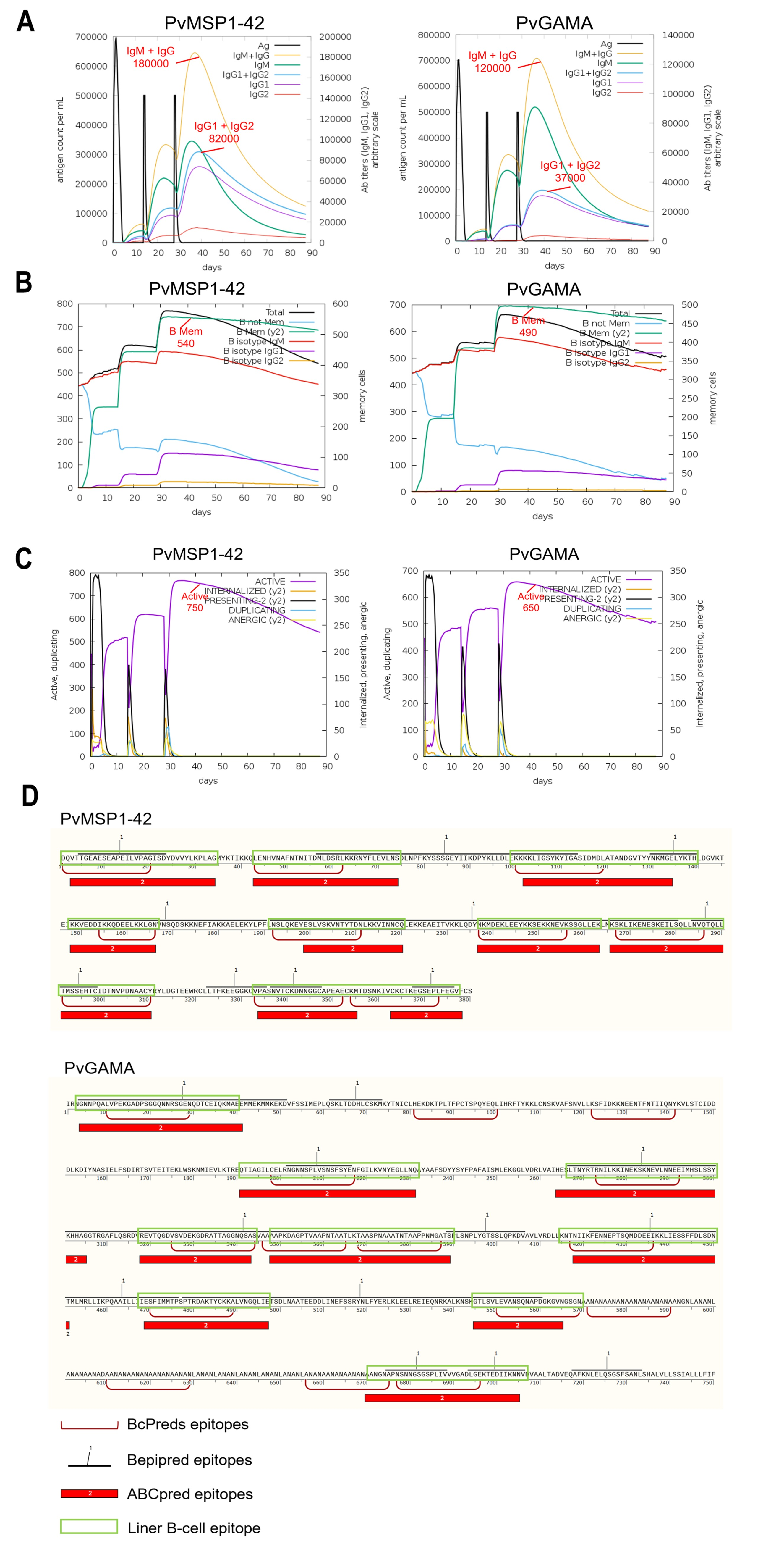

Supplement: S3 Fig — (A) The simultaneous display of both relative antibody responses and antigen concentration, along with the presence of IgG, serves as evidence of immunogenicity of the two proteins. (B) B cell population (cells/mm3) and (C) B cell population per state (cells/mm3), these graphs described the relative number of plasma cells that produce antibodies. The value of IgM + IgG, IgG1 + IgG2, B memory cells and active B cells for day 43 was shown in red. (D) In silico prediction of linear epitopes was performed on the PvMSP1-42 and PvGAMA sequence using Bcpreds, Bepipred, and ABCpred algorithms. Green boxes indicated the predicted liner B-cell epitopes. Sequences longer than nine amino acids and predicted by at least two algorithms were considered as B-cell linear epitopes. (TIF) [file ppat.1012334.s003.tif]

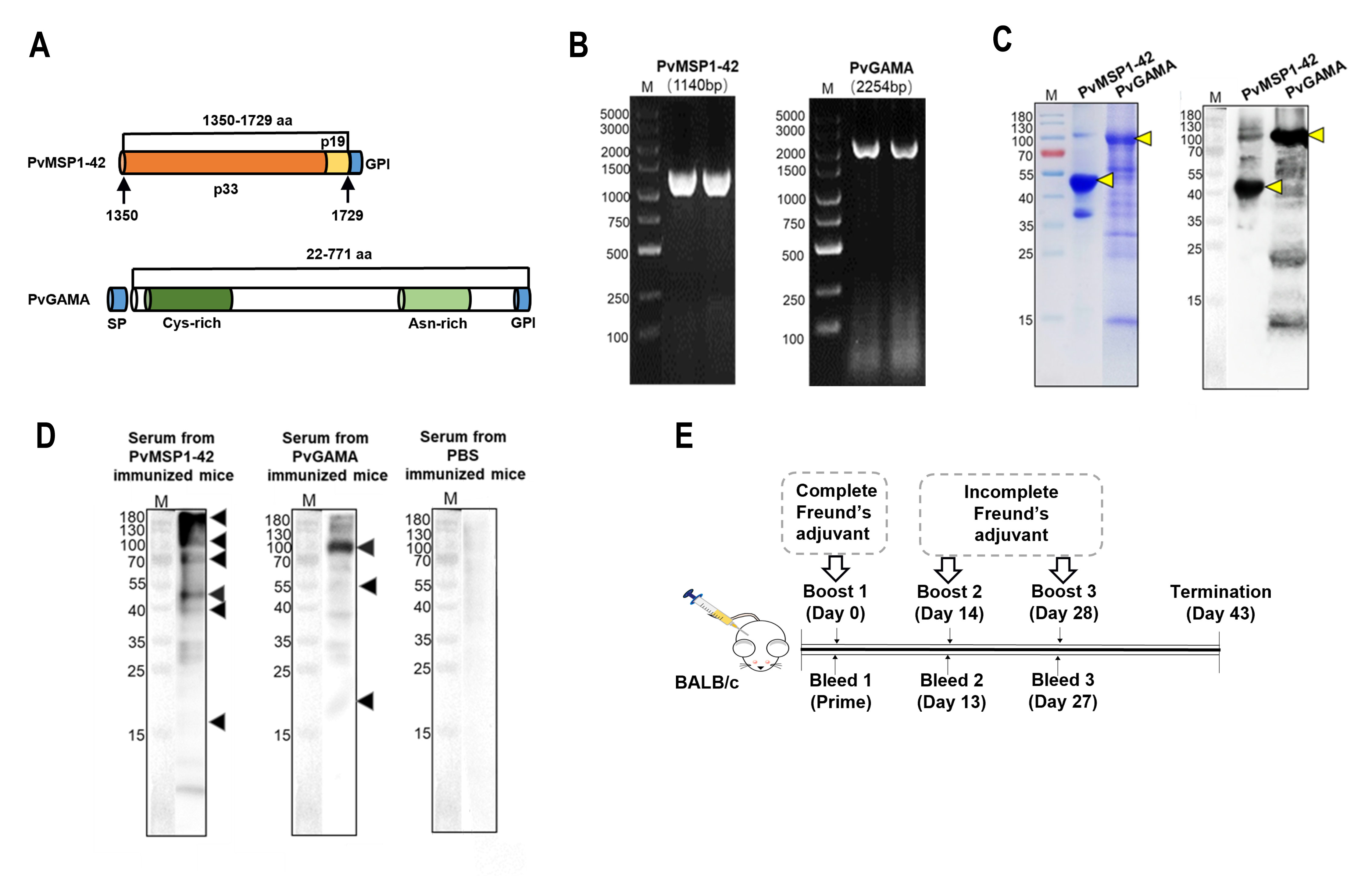

Supplement: S4 Fig — (A) Diagram of PvMSP1-42 (aa 1350–1729) and PvGAMA (aa 22–771) fragment used for recombinant protein expression. (B) Electrophoresis gels presenting the expected bands of the pvmsp1-42 (1140 bp) and pvgama (2254 bp) genes. (C) SDS-PAGE and western blot confirming the presence of PvMSP1-42 (~ 42 kDa) and PvGAMA (~ 80 kDa) (M: marker). (D) Western blot confirming the presence of PvMSP1-42 and PvGAMA in P. falciparum lysate were detected in the serum of immunized mice. Arrows represent specific bands for each recombinant protein. (E) Design of the strategy used to immunize mice. (TIF) [file ppat.1012334.s004.tif]

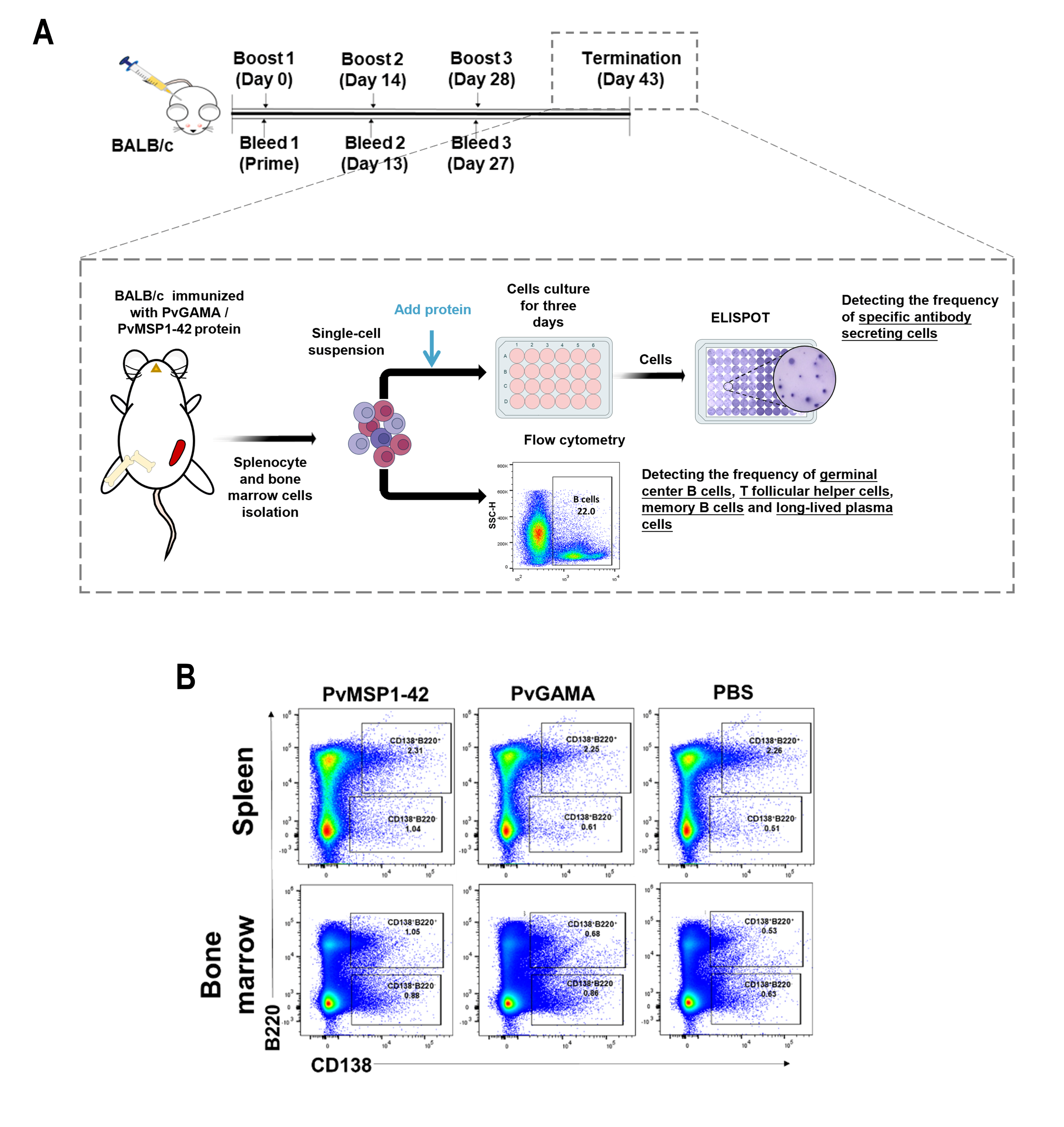

Supplement: S5 Fig — (A) The flow chart presents the strategy used to detect B cell memory formation-related cells after protein immunization in mice. (B) Representative dot plots of PBs (CD138+B220+) and PCs (CD138+B220-) detected by FACS in the spleen and the bone marrow of PvMSP1-42-, PvGAMA- and PBS-immunized mice. (TIF) [file ppat.1012334.s005.tif]

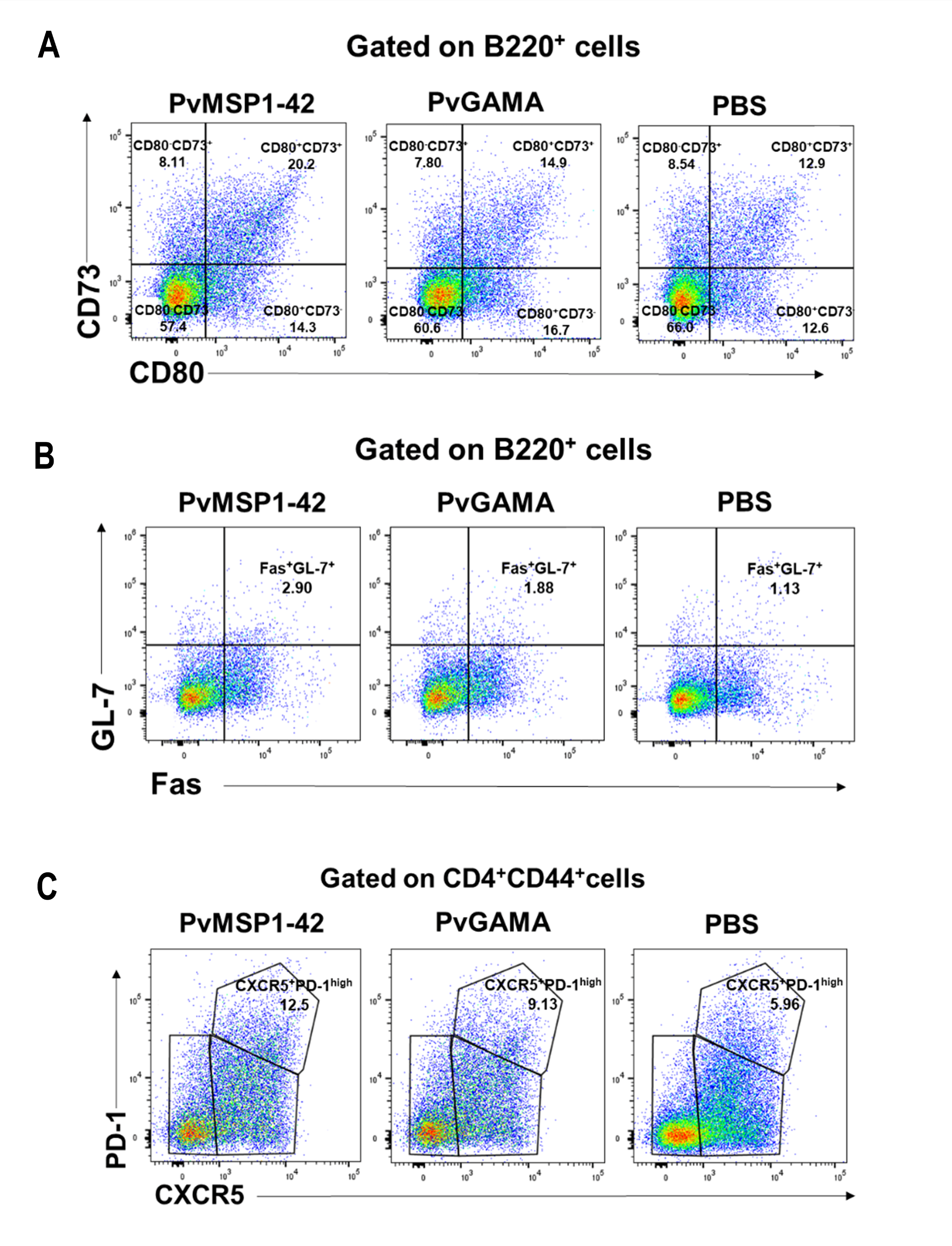

Supplement: S6 Fig — (A) Representative dot plots of CD73+CD80+ MBCs and CD73-CD80- naïve B cells on B220+ pre-gated B cells in sera from PvMSP1-42-, PvGAMA- and PBS-immunized mice. (B) Representative dot plots of splenic GC B cells (GL7+Fas+) on B220+ pre-gated B cells in sera from PvMSP1-42-, PvGAMA- and PBS-immunized mice. (C) Representative dot plots of GC Tfh cells (CXCR5+PD-1high) on CD4+ CD44+ pre-gated T cells in sera from PvMSP1-42-, PvGAMA- and PBS-immunized mice. (TIF) [file ppat.1012334.s006.tif]

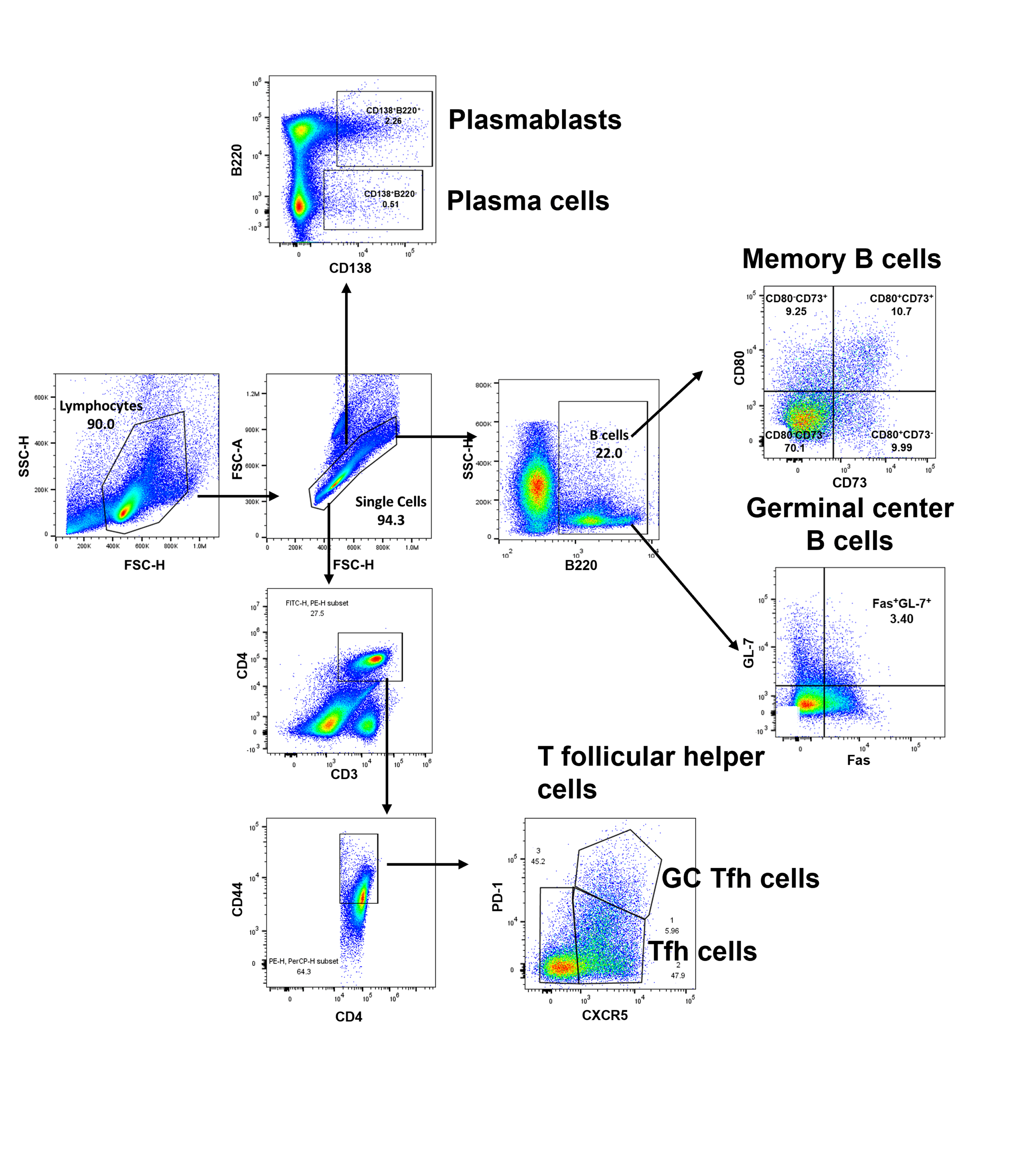

Supplement: S7 Fig — Lymphocytes were identified by forward scatter (FSC-H) vs side scatter (SSC-H) density plots. Using FSC-H and FSC-A to identify single cells, B cells were defined as B220+, memory B cells subsets of naïve and mature B cells were defined by the relative expression of CD73 and CD80 for naïve B cells (CD73-CD80-) and CD73+CD80+ memory B cell subsets, plasma cells were defined as B220-CD138+ and plasmablasts as B220+CD138+. Germinal center B cells were defined as B220+Fas+GL-7+, Germinal center T follicular helper (GC Tfh) cells as CD3+ CD4+ CD44+ CXCR5+ PD-1hi and T follicular helper (Tfh) cells as CD3+ CD4+ CD44+ CXCR5+ PD-1int. (TIF) [file ppat.1012334.s007.tif]

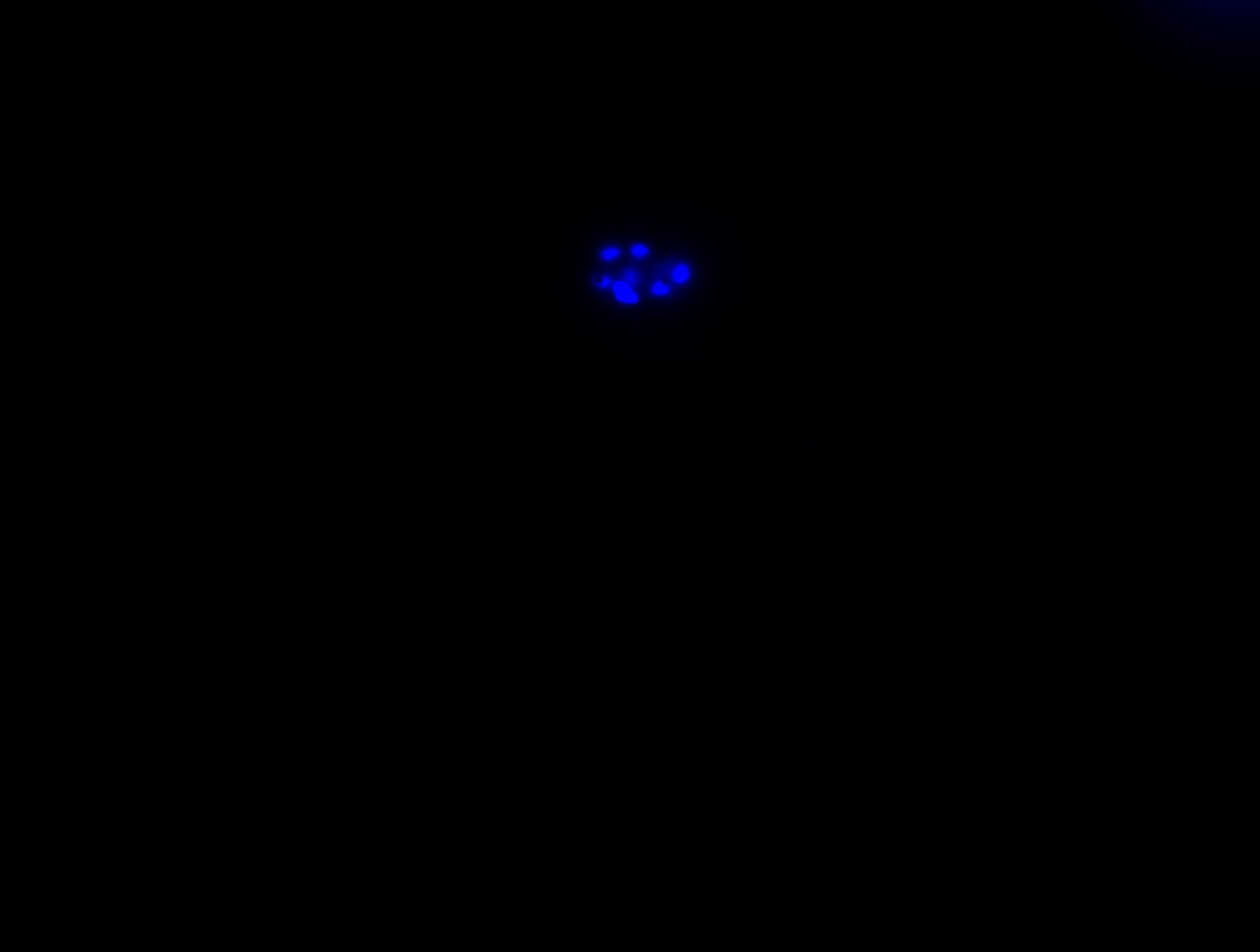

Supplement: S2 Data — (ZIP) [file ppat.1012334.s013.zip › IFA/PBS DAPI.jpg]

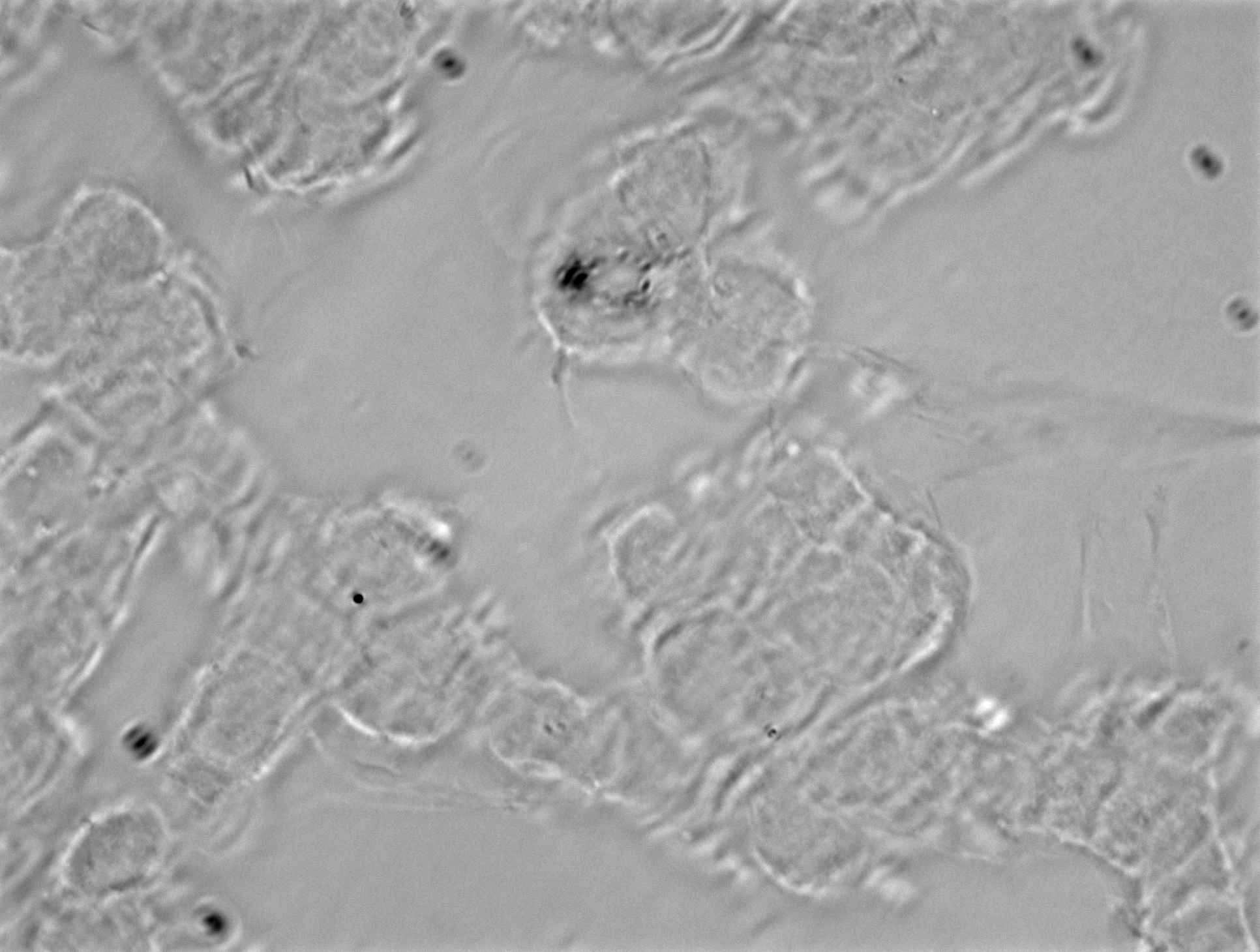

Supplement: S2 Data — (ZIP) [file ppat.1012334.s013.zip › IFA/PBS DIC.jpg]

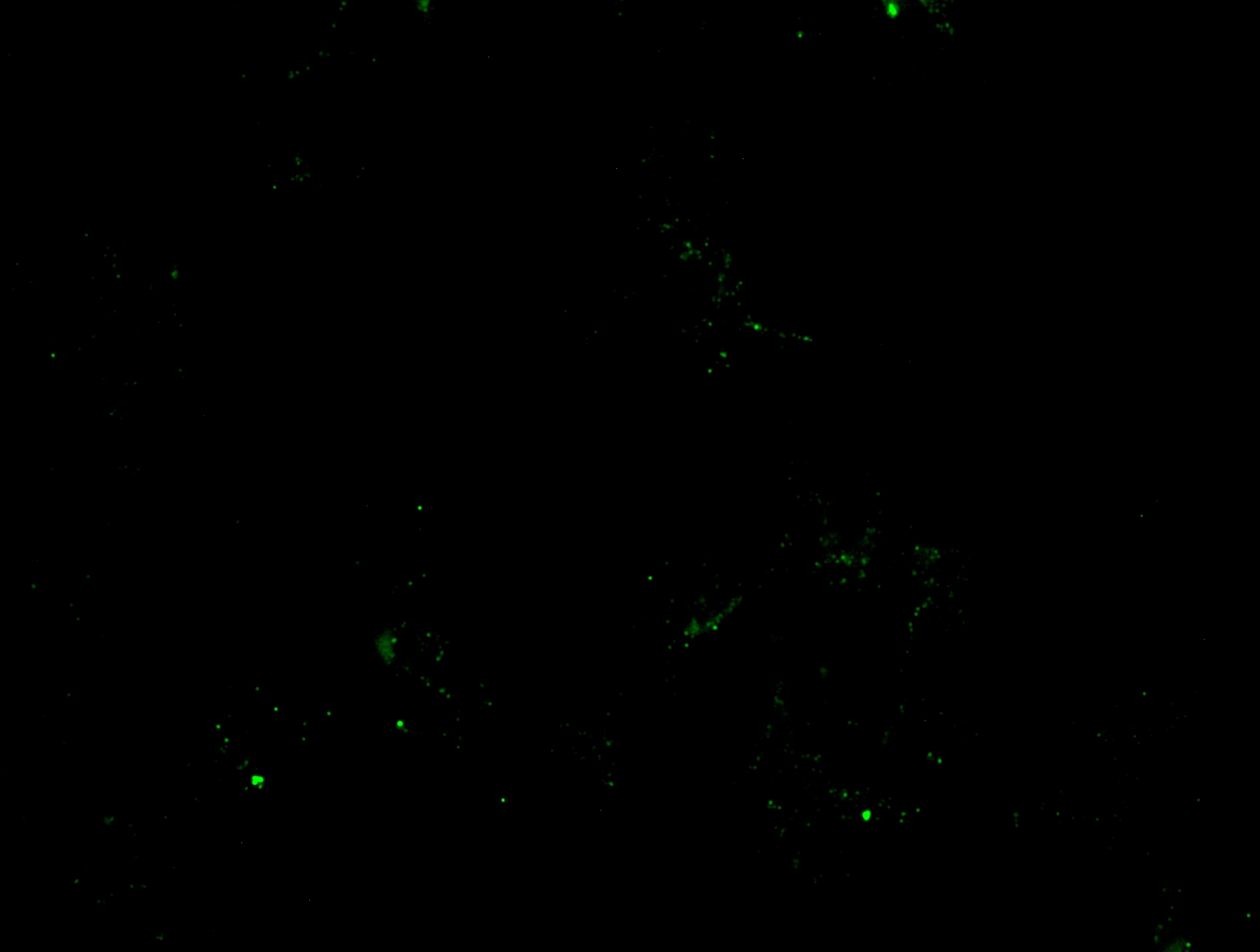

Supplement: S2 Data — (ZIP) [file ppat.1012334.s013.zip › IFA/PBS FITC.jpg]

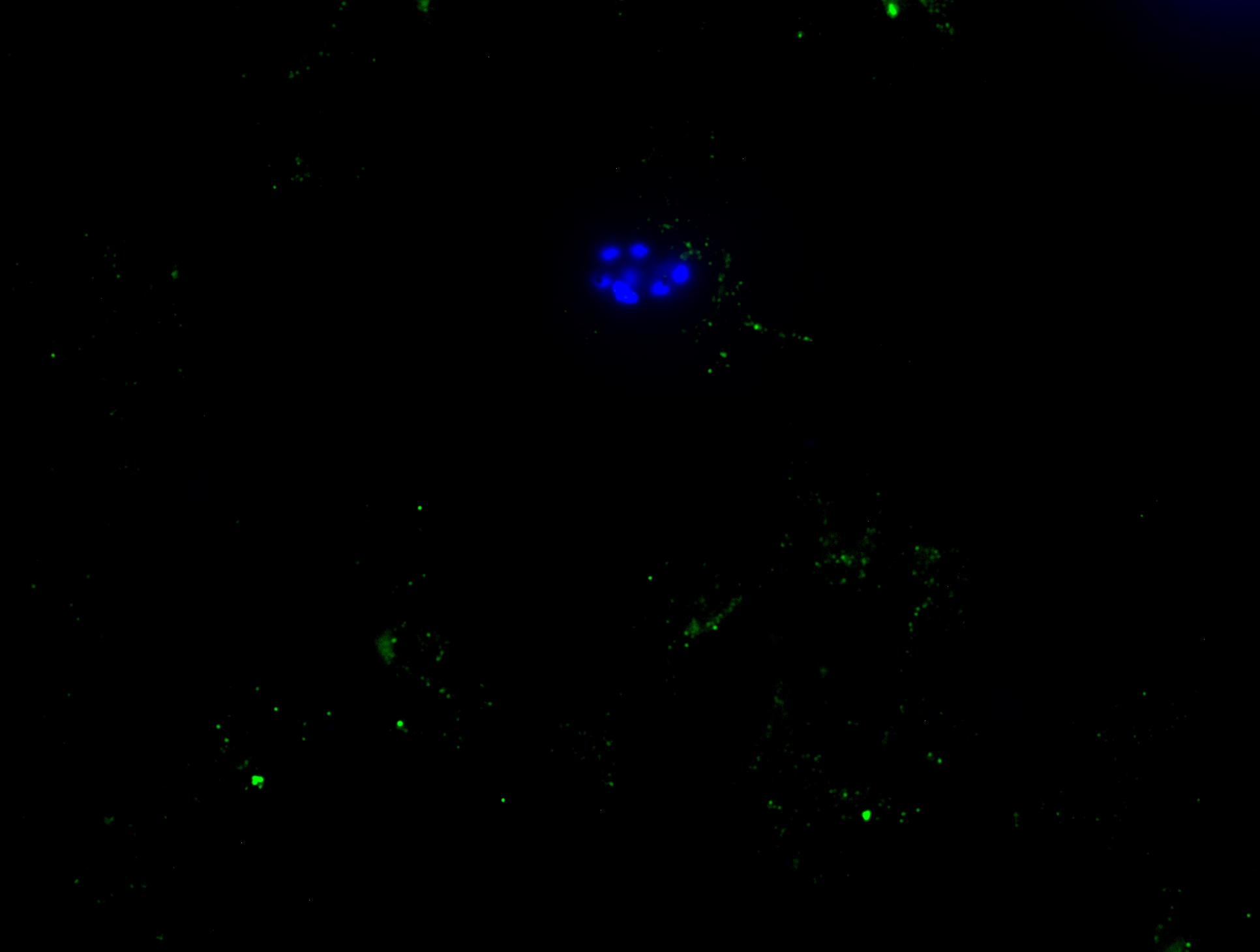

Supplement: S2 Data — (ZIP) [file ppat.1012334.s013.zip › IFA/PBS Merge.jpg]

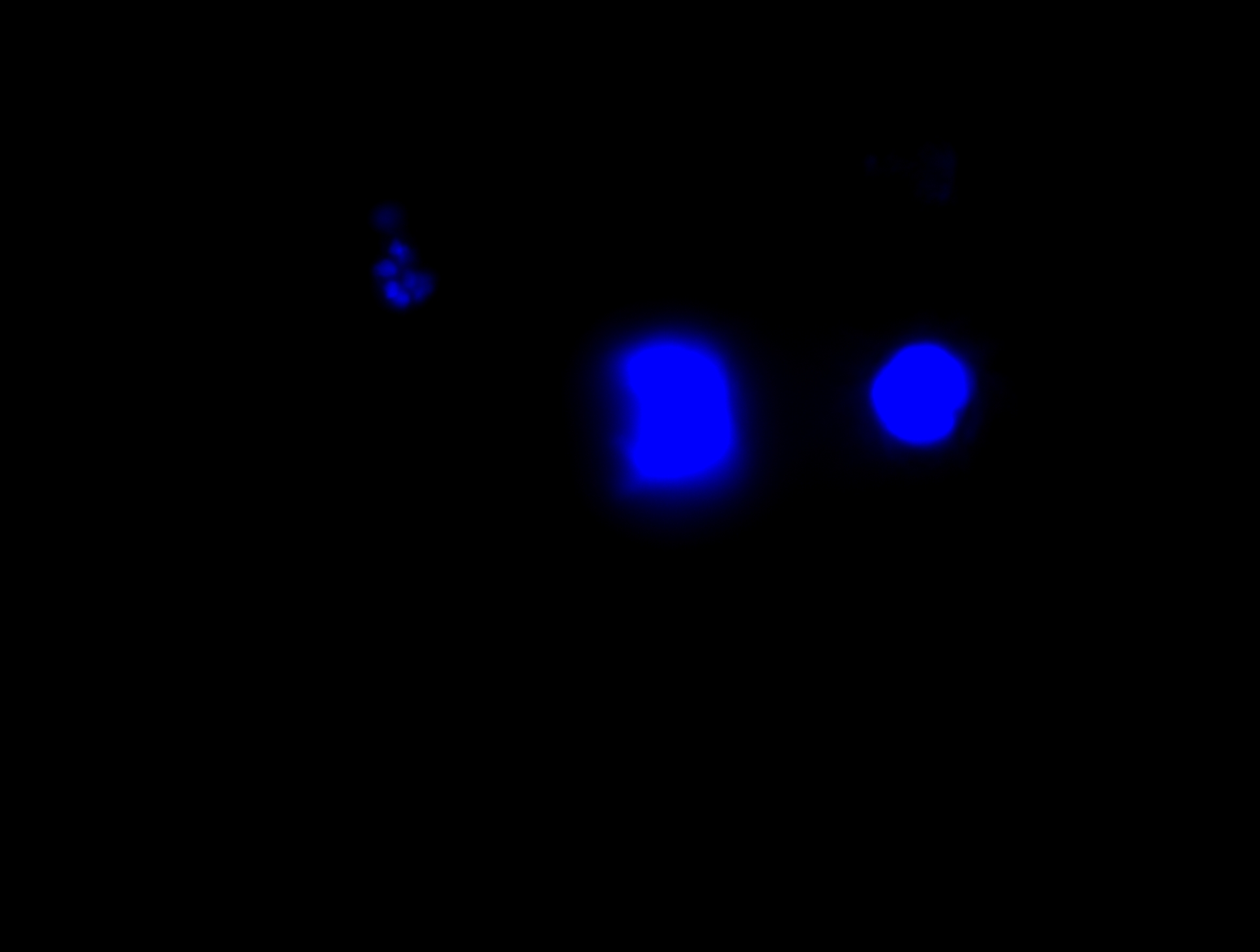

Supplement: S2 Data — (ZIP) [file ppat.1012334.s013.zip › IFA/PvGAMA DAPI.jpg]

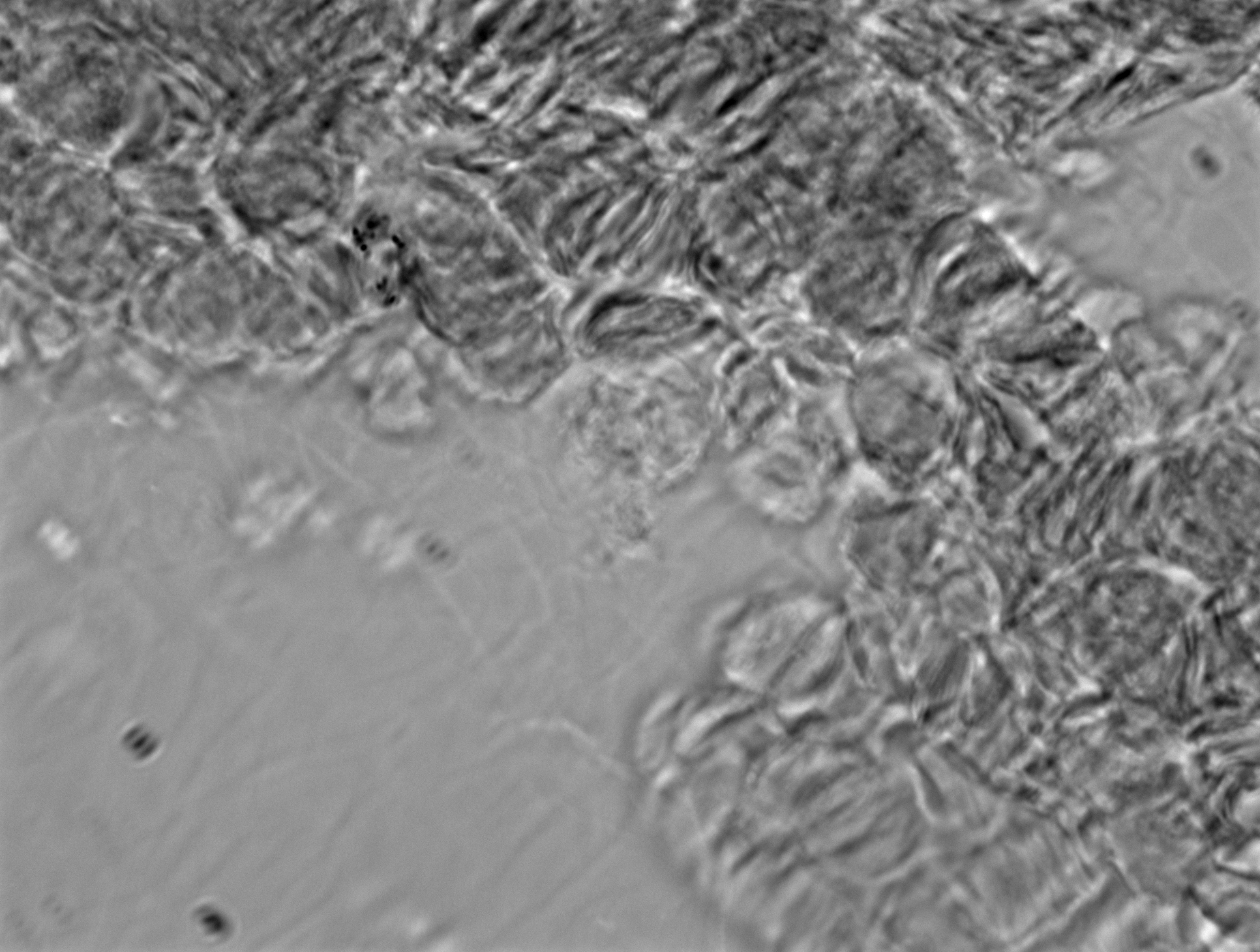

Supplement: S2 Data — (ZIP) [file ppat.1012334.s013.zip › IFA/PvGAMA DIC.jpg]

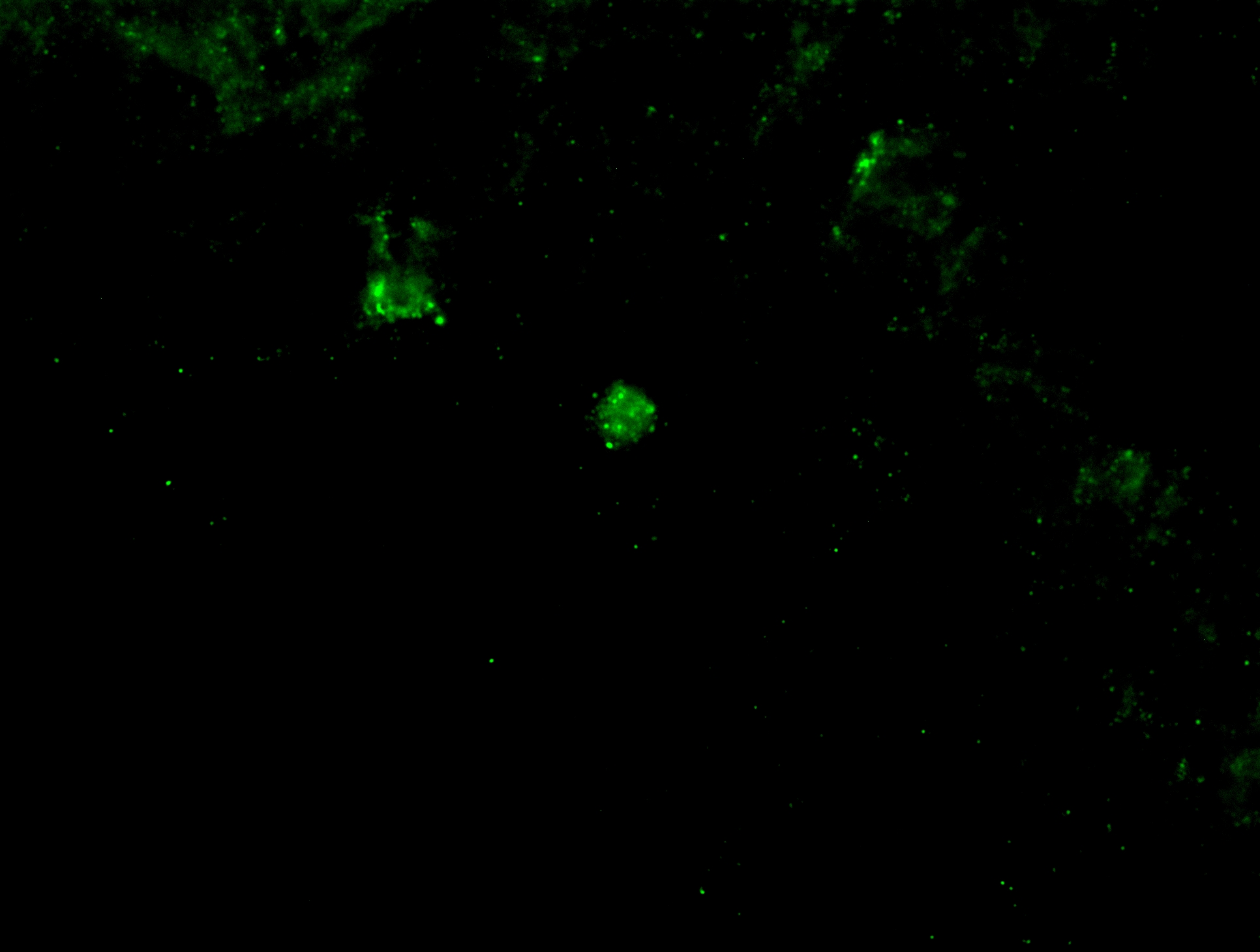

Supplement: S2 Data — (ZIP) [file ppat.1012334.s013.zip › IFA/PvGAMA FITC.jpg]

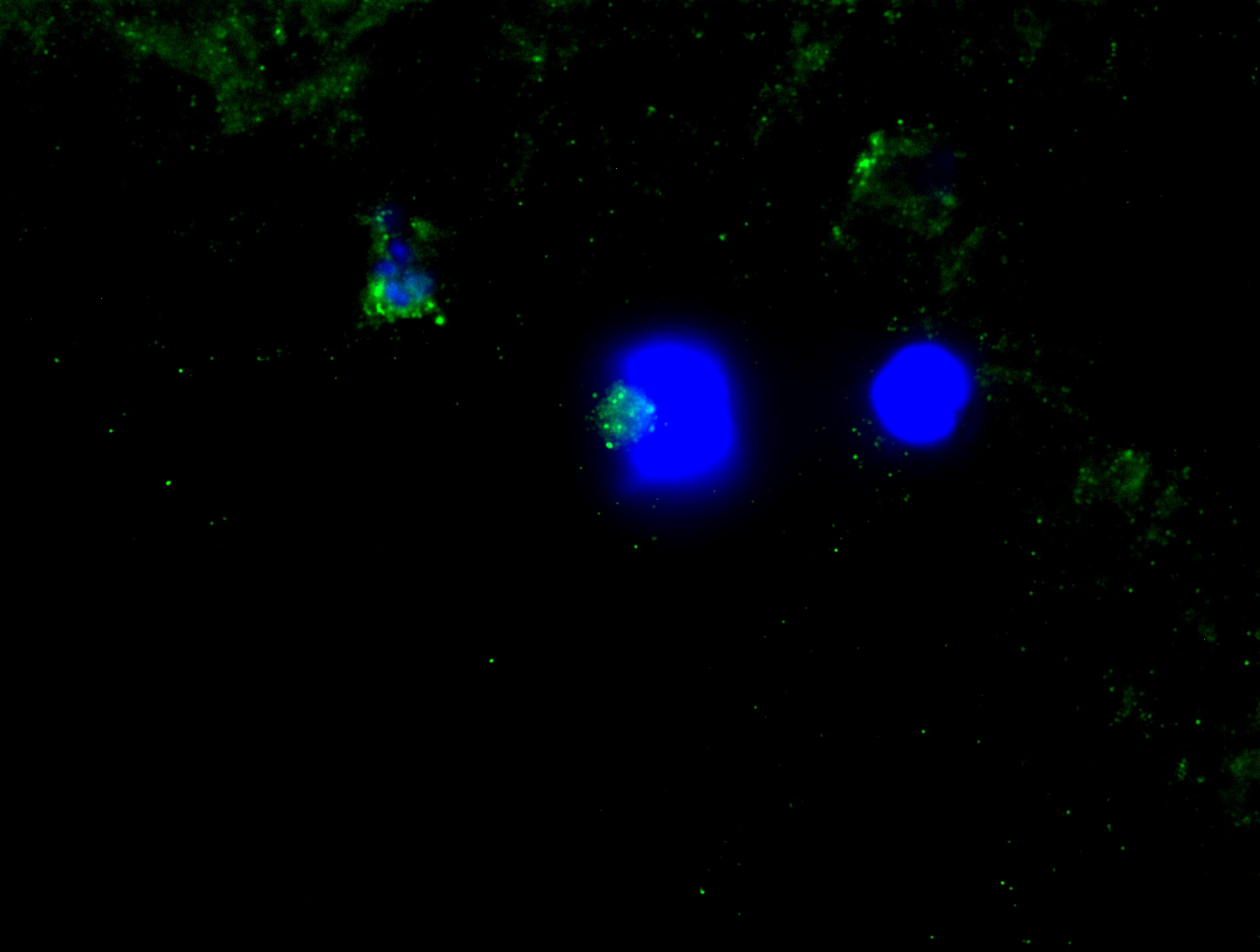

Supplement: S2 Data — (ZIP) [file ppat.1012334.s013.zip › IFA/PvGAMA Merge.jpg]

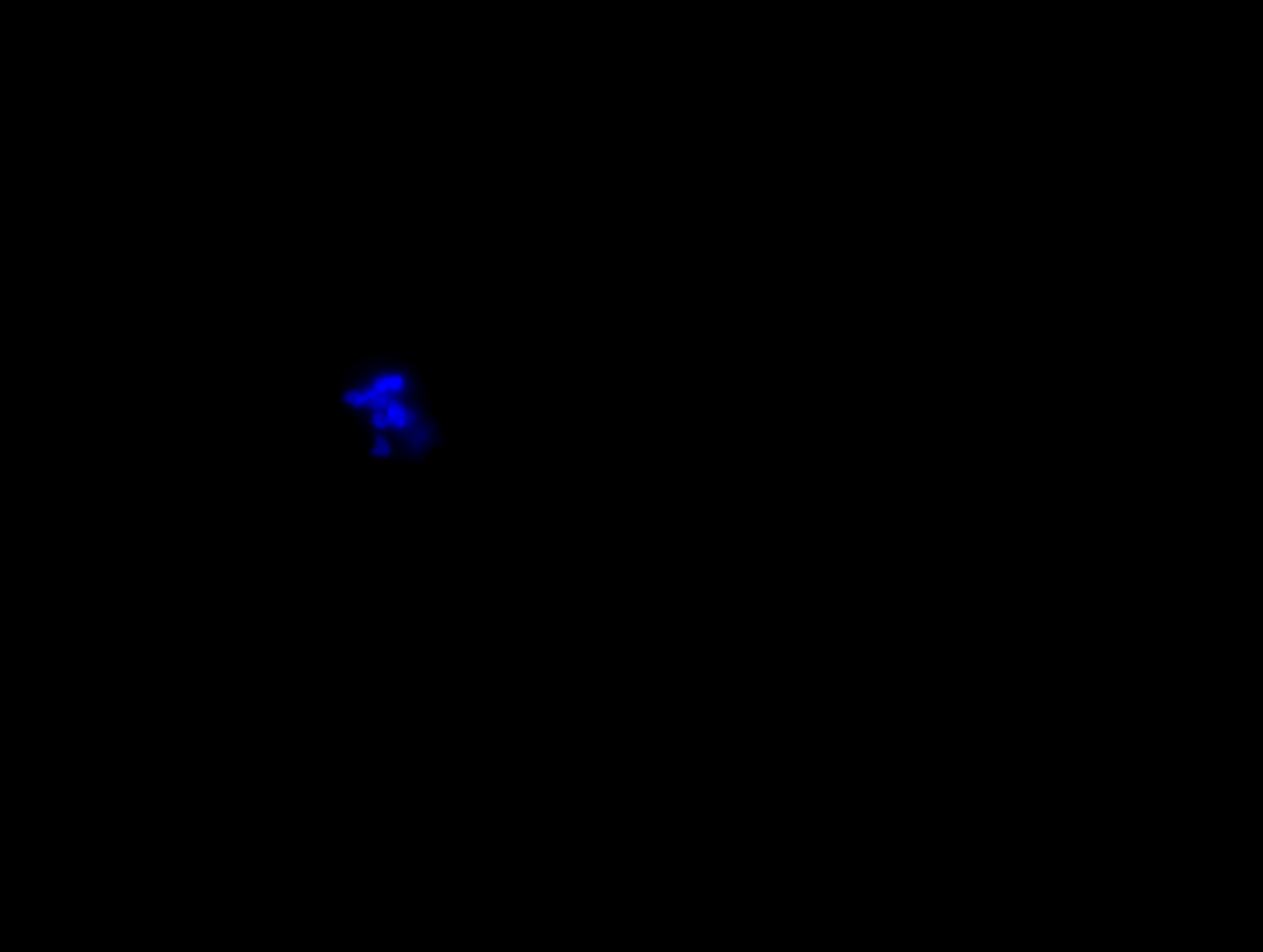

Supplement: S2 Data — (ZIP) [file ppat.1012334.s013.zip › IFA/PvMSP1-42 DAPI.jpg]

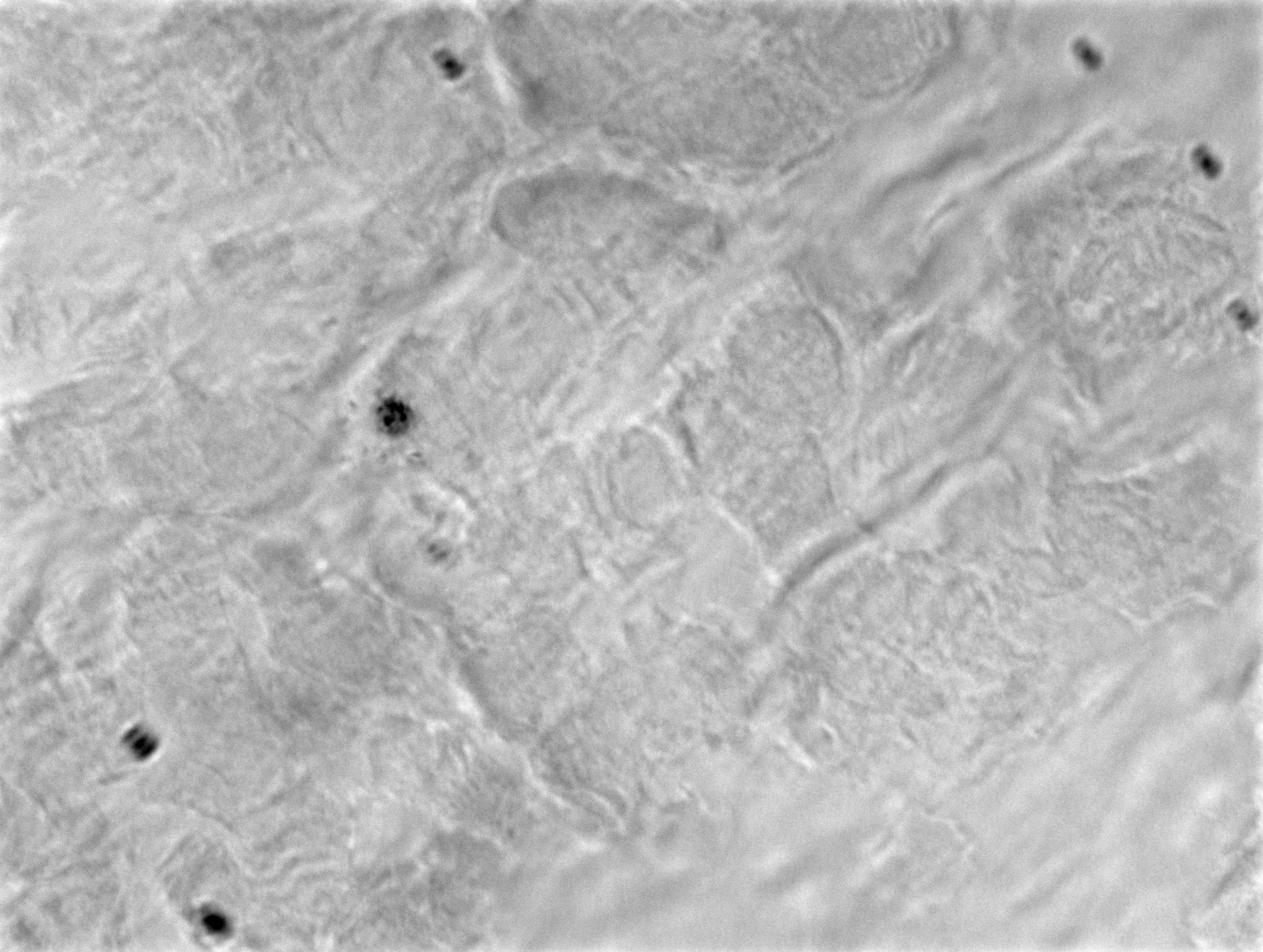

Supplement: S2 Data — (ZIP) [file ppat.1012334.s013.zip › IFA/PvMSP1-42 DIC.jpg]

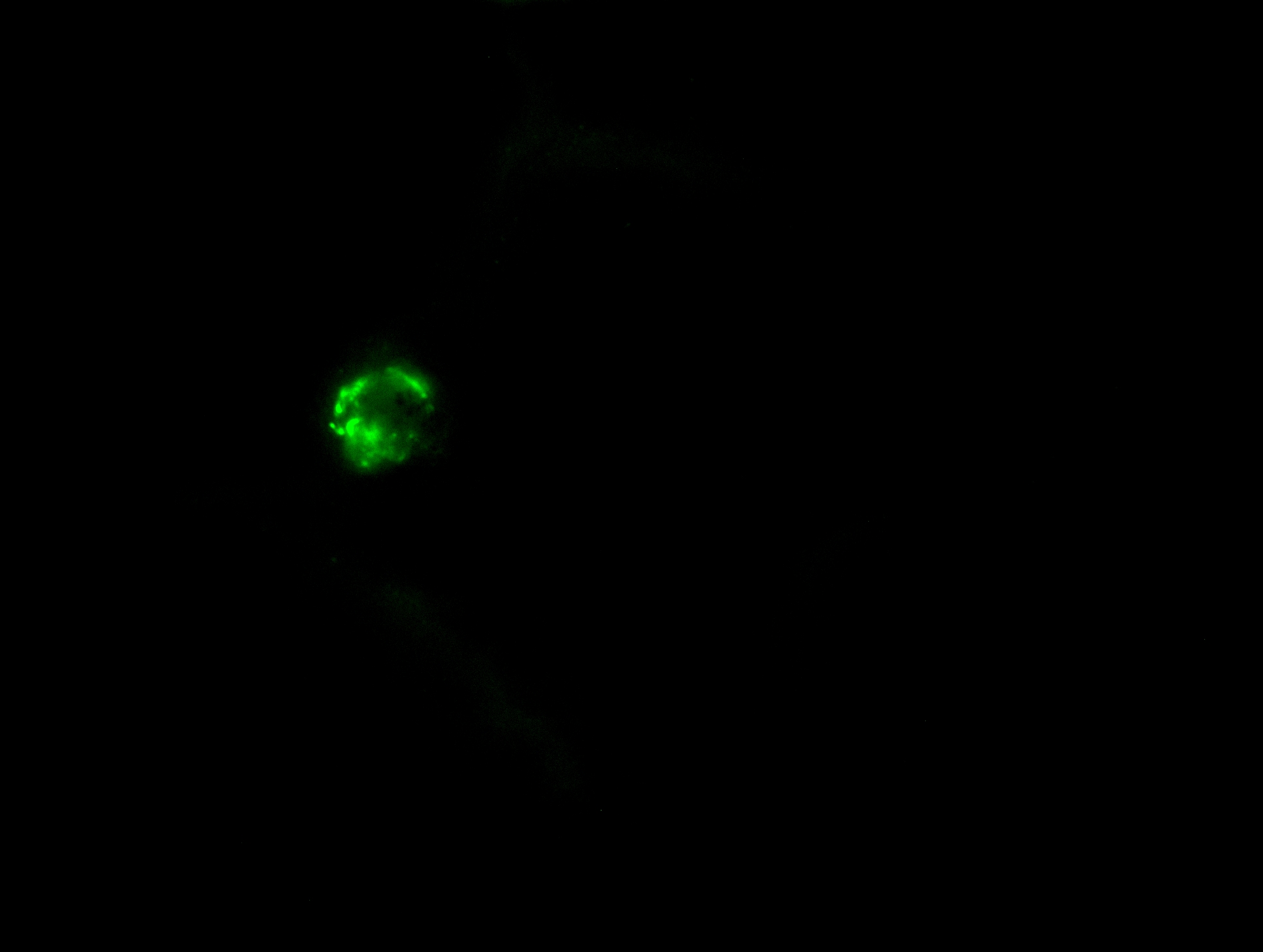

Supplement: S2 Data — (ZIP) [file ppat.1012334.s013.zip › IFA/PvMSP1-42 FITC.jpg]

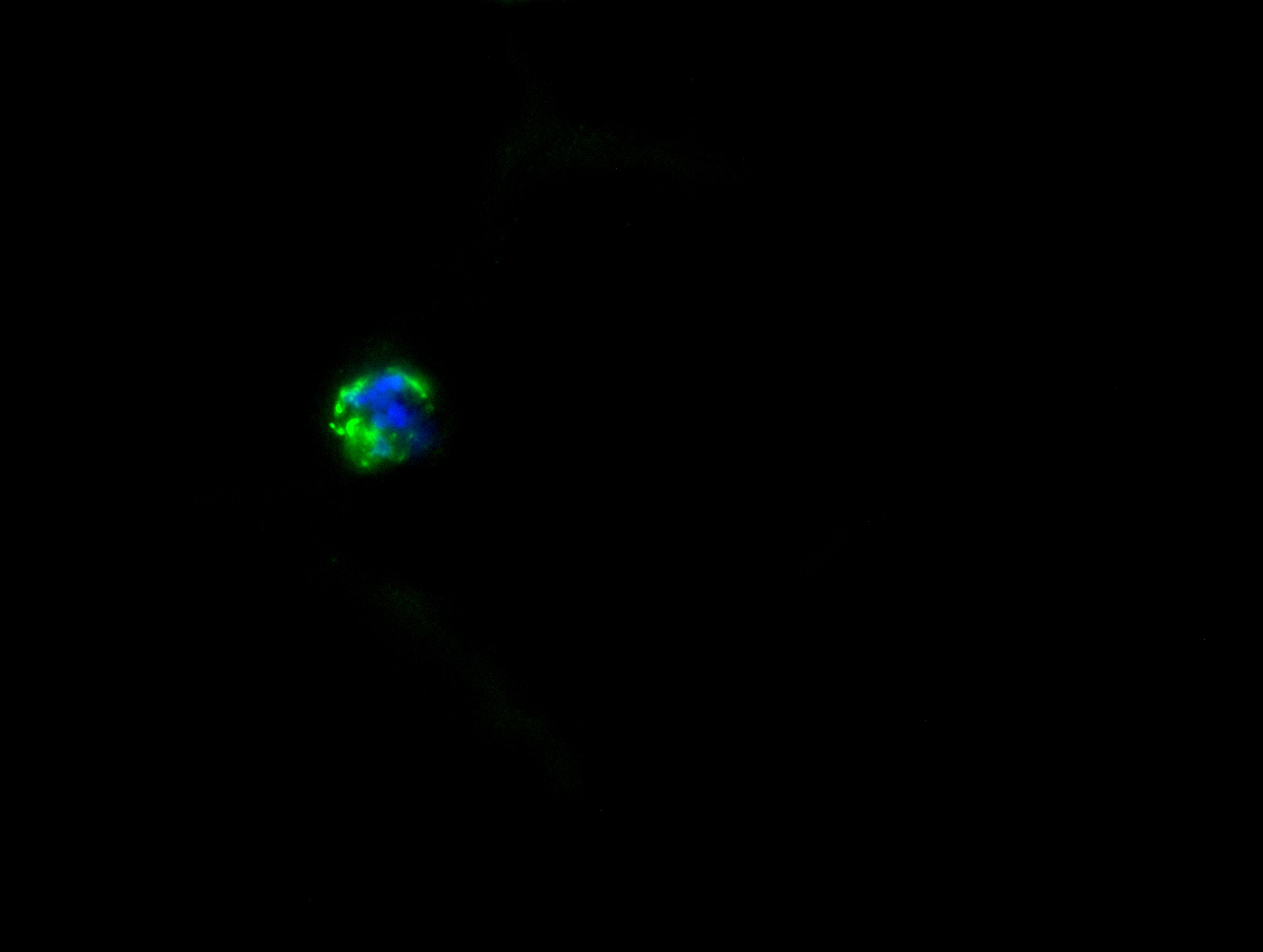

Supplement: S2 Data — (ZIP) [file ppat.1012334.s013.zip › IFA/PvMSP1-42 Merge.jpg]
