# Supplementary material for: Plasmodium vivax serological exposure markers: PvMSP1-42-induced humoral and memory B-cell response generates long-lived antibodies
Source: PLoS Pathog. 2024 Jun 28;20(6):e1012334. doi: 10.1371/journal.ppat.1012334 (PMC11239109; doi:10.1371/journal.ppat.1012334)
Supplement: S1 Table — (PDF) [file ppat.1012334.s008.pdf]

S1 Table. Classification of putative antigenic proteins on predicted subcellular function or location.

Class description	No. of		Predicted subcellular location/function
	genes	fragments	
Hypothetical protein	74	82	Not known
Other well-known	31	33	Antigenicity proteins
Enzyme	18	18	Metabolic process
GPI-anchored	16	20	Apical membrane protein or merozoite surface/cell invasion
RBP family	7	8	Host cell selection and antigenic variation
SERA family	3	3	Protein folding
Exported proteins	10	10	Parasitophorous vacuole membrane
Rhoptry protein	6	8	Rhoptry/cell invasion
Plasmodium membrance protein	16	16	Plasmodium membrance/hemoglobin catabolic process
Variable surface protein	5	5	Immune evasion
Merozoite surface protein	20	21	Immunogenic proteins
Pv-fam-d	4	4	Not known
Total	210	228	/
