# Supplementary material for: Plasmodium vivax serological exposure markers: PvMSP1-42-induced humoral and memory B-cell response generates long-lived antibodies
Source: PLoS Pathog. 2024 Jun 28;20(6):e1012334. doi: 10.1371/journal.ppat.1012334 (PMC11239109; doi:10.1371/journal.ppat.1012334)
Supplement: S2 Table — (PDF) [file ppat.1012334.s009.pdf]

S2 Table. Comprehensive screening of the 11 *P.vivax* proteins with different malaria historic individual sera/Prevalence of IgG antibodies against the 11 *P. vivax* proteins.

Antigen	Sample	No. positive	No. negative	Sensitivity [a] (%)	Specificity [b] (%)	Mean fluorescence intensity (MFI)		*P* value
						MFI±SEM	Highest MFI<br>Lowest MFI	
**PVX_088910 (GAMA)**	AVM	19	1	95.0		13,829±2,740	40,856<br>991	< 0.0001
	5-y-RI	8	29	21.6		2,387±698	25,290<br>547	0.149
	12-y-RI	6	24	20.0		1,195±143	4,376<br>451	0.149
	30-y-RI	3	27	10.0		1,099±100	3,193<br>454	0.204
	HI	1	19		95.0	989±77	1921<br>598	
**PVX_099980 (MSP1-42)**	AVM	19	1	95.0		17,280±1,496	28,128<br>1,874	< 0.0001
	5-y-RI	23	14	78.4		3,536±341	8,564<br>1,049	< 0.0001
	12-y-RI	28	2	93.3		2,674±179	5,727<br>1,635	< 0.0001
	30-y-RI	17	13	56.7		2,022±160	4,207<br>1,010	0.0001
	HI	1	19		95.0	1,150±84	2,056<br>506	
**PVX_098582 (RBP1b)**	AVM	18	2	90.0		3,346±380	6,884<br>1,210	< 0.0001
	5-y-RI	19	18	51.4		1,694±117	3,274<br>633	< 0.0001
	12-y-RI	23	7	76.7		1,892±152	5,476<br>1,151	< 0.0001
	30-y-RI	10	20	33.3		1,446±121	3,319<br>780	0.004
	HI	1	19		95.0	981±38	1,334<br>655	
**PVX_097745 (ADF1)**	AVM	16	4	80.0		6,676±825	16,517<br>2,482	< 0.0001
	5-y-RI	19	18	51.4		3,712±252	6,230<br>1,031	0.0001
	12-y-RI	14	16	46.7		3,491±234	6,895<br>1,932	0.0002
	30-y-RI	12	18	40.0		3,514±306	8,829<br>1,661	0.002
	HI	1	19		95.0	2,241±142	2,799<br>820	

S2 Table (continued)

Antigen	Sample	No. positive	No. negative	Sensitivity [a] (%)	Specificity [b] (%)	Mean fluorescence intensity (MFI)		*P* value
						MFI±SEM	Highest MFI<br>Lowest MFI	
**PVX_117880 (RON2)**	AVM	18	2	90.0		5807±670	14,045<br>2,213	< 0.0001
	5-y-RI	11	26	29.7		3057±262	8,428<br>1,347	0.011
	12-y-RI	10	20	33.3		2775±202	6,281<br>1,426	0.013
	30-y-RI	11	19	36.7		2842±253	7,389<br>1,467	0.028
	HI	1	19		95.0	2088±123	3,253<br>1,370	
**PVX_079980 (HP)**	AVM	17	3	85.0		7,233±886	19,088<br>2,960	< 0.0001
	5-y-RI	10	27	27.0		3,427±282	9,312<br>1,326	0.084
	12-y-RI	17	13	56.7		4,049±304	10,677<br>2,280	0.002
	30-y-RI	7	23	23.3		3,288±228	7,031<br>1,975	0.070
	HI	7	13		65.0	2,722±151	4,036<br>2,038	
**PVX_090945 (HP)**	AVM	17	3	85.0		6,995±869	16,750<br>2,315	< 0.0001
	5-y-RI	9	28	24.3		3,054±231	7,386<br>1,135	0.232
	12-y-RI	8	22	26.7		3,467±219	5,708<br>1,587	0.009
	30-y-RI	6	24	20.0		3,180±280	7,763<br>1,588	0.155
	HI	1	19		95.0	2,643±173	4,837<br>1,698	
**PVX_114145 (MSP10)**	AVM	19	1	95.0		10,360±1,179	21,465<br>2,314	< 0.0001
	5-y-RI	16	21	43.2		4,157±570	22,003<br>1,213	0.011
	12-y-RI	12	18	40.0		3,132±248	8,674<br>1,703	0.002
	30-y-RI	8	22	26.7		2,582±187	5226<br>1240	0.058
	HI	1	19		95.0	2,097±119	2,997<br>1,081	

S2 Table (continued)

Antigen	Sample	No. positive	No. negative	Sensitivity [a] (%)	Specificity [b] (%)	Mean fluorescence intensity (MFI)		*P* value
						MFI±SEM	Highest MFI<br>Lowest MFI	
**PVX_097715 (MSP3)**	AVM	19	1	95.0		10,140±1,107	19,801<br>2,315	< 0.0001
	5-y-RI	17	20	45.9		3,608±274	8,919<br>1,120	0.0003
	12-y-RI	17	13	56.7		3,590±257	7,848<br>1,636	< 0.0001
	30-y-RI	10	20	33.3		2,840±238	7,242<br>1,359	0.026
	HI	2	18		90.0	2,130±125	3,143<br>1,605	
**PVX_115045 (COX2a)**	AVM	17	3	85.0		8,363±1,124	25,664<br>3,813	< 0.0001
	5-y-RI	14	23	37.8		4,645±417	14,551<br>1,875	0.008
	12-y-RI	17	13	56.7		5,282±361	10,382<br>2,794	< 0.0001
	30-y-RI	13	17	43.3		4,164±307	8,051<br>1,904	0.007
	HI	0	20		100.0	3,025±185	3,829<br>1,959	
**PVX_096055 (HP)**	AVM	19	1	95.0		9,982±1,279	29,013<br>3,388	< 0.0001
	5-y-RI	16	21	43.2		4,278±371	11,236<br>1,874	0.006
	12-y-RI	7	23	23.3		3,3647±225	7,048<br>1,939	0.067
	30-y-RI	8	22	26.7		3,111±216	6,655<br>1,430	0.300
	HI	0	20		100.0	2,815±121	3,767<br>1,639	

[a] Sensitivity=Positive serum number/ Patient serum number × 100%

[b] Specificity=Negative serum number/ healthy individual serum number × 100%
