# Supplementary material for: Plasmodium vivax serological exposure markers: PvMSP1-42-induced humoral and memory B-cell response generates long-lived antibodies
Source: PLoS Pathog. 2024 Jun 28;20(6):e1012334. doi: 10.1371/journal.ppat.1012334 (PMC11239109; doi:10.1371/journal.ppat.1012334)
Supplement: S3 Table — (PDF) [file ppat.1012334.s010.pdf]

S3 Table. Physicochemical parameters and antigenicity prediction of PvMSP1-42 and PvGAMA proteins.

Protein name	Plasma DB No.	No. of amino acids (aa)	Molecular weight (kDa)	Theoretical pI	Instability index [a]	Solubility	ANTIGENpro	VaxiJen v2.0	Average antigen index
PvMSP1-42	PVX_099980	380 (1350-1729)	43.20	5.65	37.90	0.60	0.86	0.60	0.73
PvGAMA	PVX_088910	750 (22-771)	80.60	5.27	33.33	0.51	0.93	0.65	0.79

[a] An instability index threshold of less than 40 is considered stable.
