# Supplementary material for: Plasmodium vivax serological exposure markers: PvMSP1-42-induced humoral and memory B-cell response generates long-lived antibodies
Source: PLoS Pathog. 2024 Jun 28;20(6):e1012334. doi: 10.1371/journal.ppat.1012334 (PMC11239109; doi:10.1371/journal.ppat.1012334)
Supplement: S1 File — (DOCX) [file ppat.1012334.s011.docx]

**Detection of B memory related cells in mice immunized with protein**

**I. Isolation of mouse spleen and bone marrow cells**

**Materials**

1. Complete RPMI medium:

RPMI 1640 medium (Cat#11875, Solarbio), 100 U/ml penicillin, and 100 mg/ml streptomycin supplemented (Cat#P1400, Solarbio) with 10% fetal bovine serum (FBS, Cat#S9030, Solarbio)

2. 200-mesh filter (autoclave before use)

3. Sterile Petri dishes

4. Sterile syringes for single use

5. Red blood cells (RBC) lysate buffer (pH=7.4)

| Regents | MW | Amount |
| --- | --- | --- |
| NH_4_CI | 53.49 | 1.68 g |
| KHCO_3_ | 100.12 | 0.20 g |
| EDTA-Na2 | 372.24 | 0.07 g |
| ddH_2_O |  | 150 ml |
| Adjust pH to 7.4 |  |  |
| ddH_2_O |  | to 200 ml |

6. 1×Phosphate-buffered Saline (PBS, pH=7.4)

| Regents | MW | Amount |
| --- | --- | --- |
| NaCI | 58.44 | 8.00 g |
| KCI | 74.55 | 0.20 g |
| Na_2_HPO_4_ | 141.96 | 1.44 g |
| K_2_HPO_4_ | 174.20 | 0.24 g |
| ddH_2_O |  | 800 ml |
| Adjust pH to 7.4 |  |  |
| ddH_2_O |  | to 1000 ml |

**Procedures**

**1. Isolation of mouse spleen cells**

1. Fresh whole mouse spleens are minced into small pieces using sterile scissors in Petri dishes containing 10 ml of pre-cooled RPMI 1640 with 5% FBS
2. Spleen tissue pieces are gently crushed through a 200-mesh filter using a syringe plunger until the remaining spleen tissue became white.

***Note***: The movement should be gentle when grinding, too much force will cause cell death.

1. The cell suspension in Petric plate was collected into a centrifuge tube, centrifuged at 559 x*g* at room temperature for 5 min.
2. Discard the supernatant, add 5 ml RBC lysate buffer, leave the cells at room temperature for 5 min, then add 10 ml RPMI 1640 to terminate the lysate, centrifuge at 559 x*g* at room temperature for 5 min, discard the supernatant.
3. After two washes with RPMI 1640, the cells were added to 1 ml complete RPMI medium, counted, and placed on ice for subsequent use.

**2. Isolation of mouse bone marrow cells**

1. The tibia and femur muscles of the mice were separated using sterile surgical scissors and transplanted into sterile Petri dishes containing RPMI 1640.
2. The ends of the mouse femurs and tibias were cut off, and the bone marrow cavity was rinsed repeatedly with a 1 ml syringe and filtered through a 200-mesh filter into a centrifuge tube.
3. The cells were then centrifuged at 559 x*g* for 5 min, and the supernatant was discarded.
4. The pellet was resuspended in red blood cell lysis buffer and incubated for 5 min at room temperature.
5. After two washes with RPMI 1640, the cells were added to 1 ml complete RPMI medium, counted, and placed on ice for subsequent use.

**Reference**

1. Madaan A, Verma R, Singh AT et al. A stepwise procedure for isolation of murine bone marrow and generation of dendritic cells. J Biol Methods. 2015; 1(1): e1. doi:10.14440/JBM.2014.12.

2. Coquery CM, Loo W, Buszko M, Lannigan J, Erickson LD. Optimized protocol for the isolation of spleen-resident murine neutrophils. Cytometry A. 2012;81(9):806-814. doi:10.1002/cyto.a.22096

**II. Flow cytometry**

**Materials**

1. 200-mesh filter (autoclave before use)

2. 1xPBS (pH=7.4)

3. Antibodies

| Antibody/dye | Fluorochrome | Catalog # | Company |
| --- | --- | --- | --- |
| T- and B-Cell Activation Antigen (GL7) | FITC | 562080 | BD Pharmingen |
| CD45R(RA3-6B2) | APC-CY7 | 552094 | BD Pharmingen |
| CD138(281-2) | APC | 558626 | BD Pharmingen |
| CD93 (Early B Lineage) (AA4.1) | PE | 558039 | BD Pharmingen |
| CD3 | FITC | 100204 | BioLegend |
| CD4 | PE | 100408 | BioLegend |
| CD44 | Percp | 103035 | BioLegend |
| CD185 (CXCR5) | APC | 145505 | BioLegend |
| CD279 (PD-1) | PE-CY7 | 135215 | BioLegend |
| CD95 (Fas) | PE | 152607 | BioLegend |
| CD80 | PE | 104707 | BioLegend |
| CD73 | FITC | 127219 | BioLegend |

**Procedures**

1. The splenic and bone marrow cell suspension was mixed and 100 μl was sucked out of each tube for flow cytometry detection.

2. According to the cells to be measured, the corresponding antibody is added, and the concentration of each antibody is 0.8 μg/ml. Cells are incubated at 4℃ for 30 min, centrifuge at 559 x*g* for 5 min, discard the supernatant, and then wash two times with PBS. Finally, 800 μl PBS was added to re-suspend the cells.

|  | Antibody |
| --- | --- |
| Thf | CD3, CD4, D44, CXCR5, PD-1 (FITC, PE, PerCP, APC, PE-CY7) |
| GC | CD45R, GL7, Fas (APC-CY7, FITC, PE) |
| MBC | CD45R, CD73, CD80 (APC-CY7, FITC, PE) |
| LLPC | CD45R, CD138, CD93（APC-CY7, APC, PE) |

**Reference**

Bruckner, S., Wang, L., Yuan, R., Haaland, P., Gaur, A. (2011). Flow-Based Combinatorial Antibody Profiling: An Integrated Approach to Cell Characterization. In: Hawley, T., Hawley, R. (eds) Flow Cytometry Protocols. Methods in Molecular Biology, vol 699. Humana Press. https://doi.org/10.1007/978-1-61737-950-5_6

**III. B-cell EILSPOT for detecting antigen-specific antibody-secreting cells**

**Materials**

1. polyvinylidene fluoride 96-well plates

2. β-mercaptoethanol (Cat#60-24-2, Sigma)

2. PvMSP1-42 and PvGAMA protein (Filter with 0.22 micron)

3. Goat anti-Mouse IgG (Cat#SPA131, Solarbio)

4. Bovine Serum Albumin (BSA, Cat# ST023, Beyotime)

5. Biotin-highly labeled Goat Anti-mouse IgG(H+L) (Cat#A0288, Beyotime)

6. Alkaline Phosphatase-labeled Streptavidin (AP-Streptavidin, Cat# A0312, Beyotime)

7. SIGMAFAST BCIP/NBT (Cat#B5655, Sigma)

**Procedures**

1. **In vitro B-cell restimulation**

RBCs-depleted splenocytes and bone marrow cells were cultured at 5×10^5^ cells/ml in complete RPMI medium with 50 μM β-mercaptoethanol at 37°C for 3 days in the presence or absence of 25 μg/ml of PvMSP1-42 and PvGAMA protein.

1. **Prepare the ELISPOT plate (sterile conditions required)**

- Pre-wet ELISPOT plates

The PVDF membrane is soaked in 35% ethanol for 2 min at room temperature to activate the PVDF membrane. Wash plates four times by adding 200 μl sterile deionized water, and then washed twice by PBS. After discarding the liquid for the last time, gently dry it on sterile tissue.

- Protein coating

The coated proteins are diluted to 10μg/ml with PBS, added 100μl to each well, and incubated at 4 ℃ overnight. Polyclonal goat anti-mouse IgG Ab is for the total IgG ELISPOT.

1. **Cells cultured in ELISPOT plates (sterile conditions required)**

- Removing protein

Remove the protein in the plate, clean the plate five times with PBS, each time 200 μl per well, and gently dry the plate on the aseptic tissue after discarding the liquid for the last time.

- Blocking

Add 200 μl 1% BSA per well, incubate at room temperature for 2h. After incubation, wash with PBS for three times, 200 μl per well each time, discard the liquid for the last time, and gently dry on sterile paper.

- Cells planked

The stimulated cells are added into 96-well plates with 100 μl complete RPMI medium with 50 μM β-mercaptoethanol per well and 5 × 10^5^ cells /ml. The cells are evenly spread out by patting the edge of the plates, and then cultured in an incubator at 37 ℃ and 5% CO_2_ for 20 h.

***Note:***

- Do not move the plate during cultivation, and take measures to prevent evaporation by wrapping the plate in aluminum foil
- Number of cells is not recommended to exceed 5*10^5^

1. **Cells removed and the primary antibody incubated**

- Removing the cells

The cells in plate well are removed, and then incubated with PBST (PBS with 0.05% Tween-20) at room temperature for 10 min, which could be placed on a shaker, gently shaken, and washed the well five times to completely remove cells, each time 200 μl.

***Note:*** Incomplete cell removal disrupts spot formation

- Incubate the primary antibody

Biotin-highly labeled Goat Anti-mouse IgG(H+L) is diluted with PBST at a dilution ratio of 1:2000, then added 100 μl per well, incubated at 4 ℃ overnight. After incubation, wash five times with PBST.

**5. Incubate the secondary antibody and develop spot-forming cells**

- Incubate the second antibody

AP-Streptavidin is diluted with PBST at a dilution ratio of 1:2000, 100 μl was added to each well, and incubated at room temperature for 2 h. After incubation, wash five times with PBST.

- Develop spot-forming cells

Prepare substrate solution before use. Dissolve one tablet of BCIP/NBT in 10 mL of ddH_2_O. Add 100 μl per well, until clear spots appear. Deionized water is used to terminate the color reaction. Remove all excess liquid from the plate underdrain and dry overnight on a super clean bench at room temperature away from light.

***Note:*** Residual substrate can cause stain in the hole. The plate is left in the dark overnight to dry thoroughly. Observe the count using the ELISPOT reader.

**Reference**

1. Shah HB, Koelsch KA. B-Cell ELISPOT: For the Identification of Antigen-Specific Antibody-Secreting Cells. Methods Mol Biol. 2015;1312:419-426. doi:10.1007/978-1-4939-2694-7_42

2. Janetzki S, Price L, Schroeder H, Britten CM, Welters MJ, Hoos A. Guidelines for the automated evaluation of Elispot assays. Nat Protoc. 2015;10(7):1098-1115. doi:10.1038/nprot.2015.068
